# Amyloid β and Tau Alzheimer’s disease related pathology is reduced by Toll-like receptor 9 stimulation

**DOI:** 10.1186/s40478-014-0101-2

**Published:** 2014-09-02

**Authors:** Henrieta Scholtzova, Peter Chianchiano, Jason Pan, Yanjie Sun, Fernando Goñi, Pankaj D Mehta, Thomas Wisniewski

**Affiliations:** Department of Neurology, New York University School of Medicine, ERSP, 450 East 29th Street, New York, NY 10016 USA; Departments of Neurology, Pathology and Psychiatry, New York University School of Medicine, Rm 802, Alexandria ERSP, 450 East 29th Street, New York, NY 10016 USA; Department of Immunology, New York State Institute for Basic Research in Developmental Disabilities, 1050 Forest Hill Road, Staten Island, NY 10314 USA

**Keywords:** Alzheimer’s disease, Amyloid β, Tau, Oligomer, Innate immunity, Behavior, Immunohistochemistry, Transgenic

## Abstract

Alzheimer’s disease (AD) is the most common cause of dementia, and currently, there is no effective treatment. The major neuropathological lesions in AD are accumulation of amyloid β (Aβ) as amyloid plaques and congophilic amyloid angiopathy, as well as aggregated tau in the form of neurofibrillary tangles (NFTs). In addition, inflammation and microglia/macrophage function play an important role in AD pathogenesis. We have hypothesized that stimulation of the innate immune system via Toll-like receptor 9 (TLR9) agonists, such as type B CpG oligodeoxynucleotides (ODNs), might be an effective way to ameliorate AD related pathology. We have previously shown in the Tg2576 AD model that CpG ODN can reduce amyloid deposition and prevent cognitive deficits. In the present study, we used the 3xTg-AD mice with both Aβ and tau related pathology. The mice were divided into 2 groups treated from 7 to 20 months of age, prior to onset of pathology and from 11 to 18 months of age, when pathology is already present. We demonstrated that immunomodulatory treatment with CpG ODN reduces both Aβ and tau pathologies, as well as levels of toxic oligomers, in the absence of any apparent inflammatory toxicity, in both animal groups. This pathology reduction is associated with a cognitive rescue in the 3xTg-AD mice. Our data indicate that modulation of microglial function via TLR9 stimulation is effective at ameliorating all the cardinal AD related pathologies in an AD mouse model mice suggesting such an approach would have a greater chance of achieving clinical efficacy.

## Introduction

Alzheimer’s disease (AD) is the most common cause of dementia globally [1]. AD is characterized by the presence of amyloid β (Aβ) deposits in forms of parenchymal amyloid plaques and congophilic amyloid angiopathy (CAA), as well as aggregated tau protein in the form of neurofibrillary tangles (NFTs). The most toxic species of aggregated Aβ and tau are thought to be oligomeric [2]. Inflammation is another feature of AD pathology, which is linked to the production of cytokines by activated microglia. Numerous studies dating to the early 1990’s have suggested an important role for microglia in both the formation and degradation of amyloid lesions [3,4]. The importance of inflammatory pathways affecting the function of microglia for the pathogenesis of AD is highlighted by the results of genome-wide association studies (GWAS), where many of the implicated genes have a major role in immunological processes, as well as the recent linkage to AD of a rare variant of TREM2, a gene that regulates phagocytosis and the activation state of microglia/macrophages [5,6]. Microglia play a critical role in the innate immune system of the CNS and one of the most potent ways to stimulate this system is via the Toll-like receptors (TLRs).

The primary functions of TLRs are to recognize invading microbial pathogens, including bacteria, viruses, fungi and protozoans, and to activate appropriate signaling pathways to effectively clear the threat. There are 13 distinct TLR family members currently known in mammals, of which the pathogen specificities of ten (TLR1-9 and 11) have been identified [7]. We have focused on TLR9 which binds specifically to deoxyribonucleic acids (DNA) that contain unmethylated cytosine-guanosine (CpG) sequences, which are commonly found in the genomes of prokaryotes (bacteria) and viruses, while being underrepresented in those of eukaryotes. Various CpG DNA drugs that are TLR9 agonists are safe for humans and rodents [8]. We hypothesized that type B CpG oligodeoxynucleotides (ODNs) mediated stimulation of the innate immune system may be an effective way to ameliorate AD related pathology. In a prior study using the Tg2576 AD model, which develops Aβ pathology, we have shown that this approach can reduce amyloid deposition and prevent cognitive deficits [9]. However, recent experience using immunotherapeutic approaches in AD patients suggests that for clinical benefits, it is paramount to also reduce tau related pathology [10]. Furthermore, prior studies in AD models have shown that some forms of TLR stimulation can promote an increase in tau pathology [11-16]. In the present study we sought to determine whether TLR9 stimulation by CpG ODNs can ameliorate not only Aβ related pathology but also tau related pathology, while concomitantly reducing oligomer levels in the 3xTg-AD mouse model. We tested our approach by starting treatment both prior to and after the onset of pathology. We have also conducted acute TLR9 stimulation studies to help elucidate the mechanisms by which this therapeutic approach can reduce AD pathology.

## Materials and methods

### Animals and treatment

The studies were performed in the homozygous triple transgenic mouse model of AD (3xTg-AD) harboring PS1_M146V_, APP_Swe_, and tau_P301L_ transgenes [17]. These mice develop an age-dependent and progressive neuropathology that includes both amyloid plaques and NFT- like pathology. The 3xTg-AD mice used were bred internally at NYU School of Medicine on a 129/C57BL6 background and were maintained on a 12 hr light/dark cycle. All mouse care and experimental procedures were approved by the Institutional Animal Care and Use Committee at the New York University School of Medicine. Animals were injected intraperitoneally (i.p.) with either the TLR9 agonist CpG ODN 1826 (2.5 mg/kg, ~63 μg) or vehicle (saline) at monthly intervals. The mice were divided into 2 study groups treated from 7 to 20 months of age, prior to onset of pathology, and from 11 to 18 months of age, when pathology is already present. There were equal numbers of males and females in each experimental group. Treatment and control groups included 15 mice per group. CpG ODN 1826 [5’-TCC ATG A*CG* TTC CTG A*CG* TT-3’ (CpG motifs in italics)], with a complete phosphorothioate backbone, was purchased from Integrated DNA Technologies. We used the same dose of CpG ODN 1826 as in our prior study, in which we stimulated the innate immune system in Tg2576 mice [9]. Controls were non-Tg 129/C57BL6 mice injected with saline on the same schedule. During the treatment animals were closely monitored for signs of toxicity, such as differences in total body weight, and after death organs were examined for signs of pathology.

### Behavioral testing

The mice underwent a battery of behavioral tests during the final month of treatment. Prior to cognitive testing, the mice were subjected to sensorimotor activity tests. These measurements were performed to verify that any CpG ODN treatment effects observed in the cognitive tasks could not be confounded by differences in sensorimotor (locomotor) abilities.

#### Locomotor activity

A Hamilton-Kinder Smart-Frame Photobeam System was used to make a computerized recording of animal activity over a designated period of time, as we have previously described [9,18,19]. Results are reported based on distance traveled (cm), mean resting time (sec), and velocity (average and maximum) (cm/sec).

#### Rotarod

The rod apparatus was used to measure forelimb and hindlimb motor coordination and balance. The animals were first habituated with two trials to reach a baseline level of performance, and subsequently the mice were tested in three trials, with 15 min intervals between trials. In each trial, mice were placed on a 3.6 cm diameter rod (Rotarod 7650 accelerating model; Ugo Basile, Biological Research Apparatus, Varese, Italy) with initial speed set at 1.5 rpm then raised every 30 s by 0.5 rpm. A soft foam cushion was placed under the rod to prevent injury from falling. The rod was cleaned with water and 30% ethanol after each session. To assess the performance, the speed of the rod was recorded when the mouse fell or inverted (by clinging) from the top of the rotating barrel.

#### Radial arm maze

Spatial learning (working memory) was evaluated using an eight-arm radial maze with a water well at the end of each arm, as we have previously reported [9,20]. Animals entered and exited all arms of the apparatus from a central area which was controlled by clear guillotine doors and operated by a remote pulley system. Prior to each testing day, the mice were adapted to the room with lights on for 15 min. After 4 days of adaptation, water-restricted mice (2 hrs daily access to water) were given one test session per day for 12 consecutive days. For each session, all arms were baited with 0.1% saccharine solution, and animals were permitted to enter all arms until the eight rewards had been consumed. The number of errors (entries to previously visited arms) and time to complete each session were recorded. The behavioral testing was performed by an individual blinded to the animal’s treatment status.

### Histological studies

Following behavioral testing, the mice were anesthetized with sodium pentobarbital (150 mg/kg, i.p.) and perfused transaortically with 0.1 M PBS, pH 7.4. The brains were removed and the right hemisphere was immersion-fixed in periodate-lysine paraformaldehyde (PLP), whereas the left hemisphere was snap-frozen for biochemical analyses (measurements of Aβ levels and oligomers) and tau protein levels (pathological and total tau). After fixation, brains were placed in 2% DMSO/20% glycerol in PBS and stored at 4°C until sectioned. Serial coronal brain sections (40 μm) were cut on microtome and ten series of sections were saved for histological and immunohistochemical analysis of staining with: (1) anti-Aβ antibodies 6E10/4G8, (2) Thioflavin-S, (3) anti-GFAP antibody, (4) anti-CD11b antibody, (5) anti-CD45 antibody, (6) anti-CD3 antibody, (7) anti-CD206, (8) anti-Iba1, (9) anti-PHF1 antibody, (10) anti-AT8 antibody, (11) anti-MC1 antibody, and (12) anti-HT7 antibody. Aβ deposits were stained either with a mixture of mouse monoclonal antibodies 6E10/4G8 (total amyloid) or Thioflavin-S for fibrillar amyloid (parenchymal and CAA). The following monoclonal antibodies MC1 (conformation dependent), PHF1 (Ser396/Ser404), AT8 (Ser202/Thr205) and HT7 (total human tau) were used to examine tau related pathology as described [18,21,22]. GFAP is a component of the glial intermediate filaments that form part of the cytoskeleton in astrocytes and is often employed as a marker of astrocytic activation. We used four different markers to identify microglia/macrophages: CD11b, CD45, CD206 and Iba1. CD11b [member of β-integrin family of adhesion molecules; also known as MAC-1 or complement receptor 3 (CR3)] and CD45 (protein-tyrosine phosphatase) are commonly used markers for the microglial activation at the earliest and later stages of plaque development, respectively [23]. CD11b labels macrophages/microglia in both the M1 and M2 activation states, while CD45 is associated with the M1 classical activation state [24,25]. CD206 [or mannose receptor 1 (MRC1)] is an immunohistological marker for the M2 state of macrophage/microglia alternative activation [24,26,27]. Iba1 (ionized calcium binding adaptor molecule) labels all microglia, whether active or resting, and has been used as generic microglial marker [28,29]. The remaining series were placed in ethylene glycol cryoprotectant (30% sucrose/30% ethylene glycol in 0.1 mol/L phosphate buffer) and stored at −20°C.

Immunohistochemistry was performed on free floating sections as previously described [9]. Briefly, free-floating sections were incubated in MOM diluents (Vector Laboratories Inc., Burlingame, CA) containing different mouse monoclonal primary antibodies at 4°C overnight [anti-Aβ antibodies 6E10 and 4G8, 1:1000 (Covance Research Products Inc., Denver, PA); anti-tau antibodies MC1, 1:100 (provided by Dr. Peter Davies); PHF1, 1:100 (provided by Dr. Peter Davies); AT8, 1:500 (Thermo Fisher Scientific Inc., Rockford, IL); HT7, 1:2000 (Thermo Fisher Scientific Inc., Rockford, IL); and T cell antibody CD3, 1:400 (Santa Cruz Biotechnology Inc., Dallas, Texas)]. GFAP [rabbit polyclonal, 1:1000; 4°C overnight (Dako Inc., Carpinteria, CA)], CD206 [rabbit polyclonal, 1:200; 4°C overnight (Abcam Inc., Cambridge, MA)] and Iba1 [rabbit polyclonal, 1:1000; 4°C overnight (Wako Chemicals, Richmond, VA)] immunostaining was performed with a primary antibody diluent composed of 0.3% Triton X-100, 0.1% sodium azide, 0.01% bacitracin, 1% bovine serum albumin (BSA), and 10% normal goat serum in PBS, and secondary biotinylated goat anti-rabbit antibody (Vector Laboratories Inc., Burlingame, CA) reacted for 1 hr at 1:1000 dilution. CD45 [rat anti-mouse, 1:1000; 4°C overnight (AbD Serotec, Raleigh, NC)] and CD11b immunohistochemistry [rat anti-mouse, 1:500; 4°C overnight (AbD Serotec, Raleigh, NC)] were performed similarly to that for GFAP staining except that the secondary antibody was goat anti-rat (Vector Laboratories Inc., Burlingame, CA) diluted 1:1000. Additionally, CD45, CD11b and CD206 staining was also performed on a separate set of brains from young (12 months) and aged (20 months) 3xTg-AD mice acutely injected (12 hours before perfusion) with CpG ODN or saline to further assess the effects of TLR9 signaling on macrophage/microglia activation. Double immunofluorescence for macrophage/microglia markers (CD45 or CD206) and Aβ deposits (6E10/4G8) or pathological tau (MC1) was done on selected sections. Secondary antibodies were Cy3-conjugated goat anti-mouse, Cy3-conjugated goat anti-rabbit, Alexa Fluor 488-conjugated goat anti-rat and Alexa Fluor 488-conjugated goat anti-rabbit [1:500; RT 2 hrs (Jackson Immuno Research Laboratories Inc, West Grove PA)]. Fluorescent imaging was performed on a Zeiss LSM 700 inverted confocal microscope. ZEN 2011 software was used for data collection and analyses. Perl’s Prussian blue staining for ferric iron in hemosiderin (a degradation product of hemoglobin) was performed on another set of sections to detect cerebral bleeding, as described previously [30]. Equally spaced sections were mounted and stained in a solution containing 10% potassium ferrocyanide and 20% hydrochloric acid for 45 min. Fifteen Perl stained sections were examined per mouse and the average number of iron positive profiles per section was calculated.

#### Image analysis - quantification of amyloid and CD45 burden

Immunostained tissue sections were quantified with a Bioquant stereology semiautomated image analysis system (R&M Biometrics) using a random unbiased hierarchical sampling scheme, as published previously [9,18,31]. Fifteen sections were analyzed per animal. All procedures were performed by an individual blinded to the experimental study. Total Aβ burden (defined as the percentage of test area occupied by Aβ) was quantified for the cortex and for the hippocampus on coronal plane sections stained with the monoclonal anti-Aβ antibodies 6E10 and 4G8. Total fibrillar Aβ burden (parenchymal and vascular) and CAA burden (Aβ burden in the vasculature) were evaluated separately in sections stained with Thioflavin-S, using methods described previously [9]. The CD45 microglia burden (the percentage of area in the measurement field occupied by CD45 immunoreactive microglia) was quantified in a manner analogous to that used to measure the Aβ burden.

#### Analysis of tau burden

Semiquantitative analysis of tau burden in brain sections was based on the severity of neuronal PHF1, AT8, MC1 and HT7 immunoreactivity as described previously [18,19]. Approximately thirteen cortical and seven hippocampal sections were analyzed per animal at 10 × magnifications. Cortical immunolabeling was minimal. The rating was based on the number of reactive neuronal perikarya and processes. Immunostained sections were evaluated on scales devised by blinded individual, which ranged from 0 to 3 on the basis of pathology intensity.

#### Rating of microgliosis

The assessment of the CD11b, CD206 and Iba1 immunostained sections was based on a semiquantitative analysis of the extent of microgliosis (0, a few resting microglia; 1, rare ramified and/or phagocytic microglia; 2, a few ramified/phagocytic microglia; 3, a moderate ramified/phagocytic microglia; 4, numerous ramified/phagocytic microglia), as we have reported previously [9,18]. Approximately thirteen cortical and seven hippocampal sections were analyzed per animal.

#### Rating of astrocytosis

Reactive astrocytosis was rated on a scale of 0–4. The rating was based on a semiquantitative analysis of the extent of GFAP immunoreactivity (number of GFAP immunoreactive cells and complexity of astrocytic branching), as we have previously published [19,30]. Approximately thirteen cortical and seven hippocampal sections were analyzed per animal by an investigator who was blinded to treatment group assignment.

### Tissue homogenization for biochemical analyses

Before extraction of Aβ and tau from brain tissue, 10% (w/v) homogenates were prepared in tissue homogenization buffer (20 mM Tris base, pH 7.4, 250 mM sucrose, 1 mM EDTA, 1 mM EGTA) with 100 mM phenylmethylsulphonyl fluoride, protease inhibitors [protease inhibitors cocktail Complete (Roche Diagnostic GmbH, Mannheim, Germany) plus pepstatin A (Sigma-Aldrich Inc., St. Louis, MO)] and phosphatase inhibitors (1 mM NaF, 1 mM Na_3_VO_4_ and 0.5 mM okadaic acid) added immediately before homogenization, as we have previously published [18,19]. Subsequently, brain homogenates were aliquoted, frozen, and stored at −80°C until used for extraction of soluble and insoluble fractions of both Aβ and tau, and their biochemical analyses (ELISA, Western blot). All quantitative biochemical analyses were performed in brain homogenate fractions of the entire left hemisphere.

### Assessment of Aβ pathology in the brain

#### Sandwich ELISA for Aβ levels

Tissue fractionation and extraction was performed as previously described [9,19]. In brief, for extraction of soluble Aβ, brain homogenates were thoroughly mixed with an equal volume of 0.4% diethylamine (DEA)/100 mM NaCl, then spun at 135 000 × g for 1 hr at 4°C, and subsequently neutralized with 1/10 volume of 0.5 M Tris, pH 6.8. Similarly for extraction of the total Aβ, homogenates (200 μl) were added to 440 μl of cold formic acid (FA) and sonicated for 1 min on ice. Subsequently, 400 μl of this solution was spun at 100,000 × g for 1 hr at 4°C. Then, 210 μl of the resulting supernatant was diluted into 4 ml of FA neutralization solution (1 M Tris base, 0.5 M Na_2_HPO_4_, 0.05% NaN_3_).

The total and soluble Aβ levels were measured using a combination of mouse monoclonal antibody 6E10 (specific to an epitope present on amino acid residues 1–16 of Aβ) and two different rabbit polyclonal antibodies specific for Aβ40 (R162) and Aβ42 (R165), in an antibody sandwich ELISA as described previously [9]. The assay was performed by an investigator blinded to group assignment.

#### Western blot analysis of Aβ oligomers

For Western immunoblot analysis, 10% (w/v) brain homogenates were centrifuged at 20,000 × g for 20 min at 4°C and the supernatants were transferred to clean tubes and stored as previously described [18,30]. The total protein concentration in the supernatant was determined using the Bicinchoninic acid assay (BCA; Pierce Biotechnolgy, Rockford, IL). Samples (25 μg total protein), mixed with an equal volume of Tricine sample buffer, were electrophoresed on 12.5% Tris-tricine polyacrylamide gels (under non-reducing conditions) and transferred to nitrocellulose membranes. The blots were blocked with 5% non-fat dry milk in Tris-buffered saline 0.05% Tween 20 (TBS-T) for 2 hrs at room temperature. Oligomer-specific A11 polyclonal antibody (Invitrogen Corporation, Frederick, MD) was diluted (1:1000) in 0.1% BSA/TBS-T and incubated with the blots for 2 hrs at room temperature (RT). Bound antibody was visualized with horseradish peroxidase-conjugated goat anti-rabbit IgG (1:3000; 1 h, Pierce Biotechnology, Rockford, IL) and the ECL detection system (Pierce Biotechnology, Rockford, IL). The specificity of A11 staining was confirmed by probing the membrane with anti-Aβ monoclonal antibodies 6E10 or 4G8 [32]. Densitometric analysis of A11 immunoreactive oligomer specific bands was performed with NIH Image J version 1.47 software.

#### Sandwich ELISA for oligomeric Aβ

The levels of Aβ oligomers were determined using the Human Aggregated Aβ ELISA kit (Invitrogen Corporation, Frederick, MD) according to the manufacturer’s instructions and as we have previously published [18,19]. In brief, oligomeric Aβ levels in each sample were measured against a standard containing aggregated Aβ. 10% brain homogenates (BH) were centrifuged at 100,000 × g for 1 hr at 4°C. Samples diluted in the provided standard diluent buffer (1:5 dilutions) were incubated for 2 hrs at RT allowing the Aβ to bind the capture antibody (a monoclonal antibody specific for the N-terminus of human Aβ pre-coated to each well), followed by extensive washing and incubation for 1 hr at RT with biotin conjugated detection antibody (same as the capture antibody) which binds to the immobilized aggregated Aβ. After removal of excess antibody, horseradish peroxidase-labeled streptavidin (SAV-HRP) was added to incubate for 30 min, followed by washing, and a tetramethylbenzidine (TMB) substrate incubation to produce a colorimetric solution. The intensity of this colored product was directly proportional to the concentration of oligomeric Aβ in the sample. The standards provided a linear curve and the best-fit line determined by linear regression were used to calculate the concentration of Aβ oligomers in samples.

### Assessment of Tau pathology in the brain

#### Western blot analysis of phosphorylated and total tau

Standard Western blot analysis was first performed on brain extracts (10% BH) that represents the supernatant (S1) obtained after centrifuging the brain homogenate at low speed (20,000 × g for 20 min). Proteins of different solubility were further extracted from brains in buffers of increasing stringency. The brain homogenates were subjected to DEA and FA extraction as described above. High speed supernatants of DEA extraction were collected and used for Western blot analysis of DEA soluble fraction. Insoluble proteins were extracted with FA (FA fraction) [33]. Protein concentration of 10% BH and both fractions were determined using bicinchoninic acid assay (BCA; Pierce Biotechnology, Rockford, IL). Equal amount of protein (20 μg) was loaded and the samples were electrophoresed on 10% SDS-PAGE gels and transferred to nitrocellulose membranes. The blots were blocked in 5% non-fat milk with Tween-20 in TBS, and incubated with the following mouse monoclonal primary antibodies overnight at 4°C: PHF1 (Ser396/Ser404, 1:500), CP13 (Ser202, 1:500), TG5 (total human and mouse tau, 1:1000) and CP27 (total human tau, 1:1000); provided by P. Davis. Subsequently, the blots were washed and incubated for 1 hr at RT with peroxidase-conjugated goat anti-mouse IgG (1:3000; GE Healthcare UK Limited, Little Chalfont, UK). The bound antibodies were detected by enhanced chemiluminescence (ECL; Pierce Biotechnology, Rockford, IL). Densitometric analyses of the immunoreactive bands corresponding to phospho-tau and/or total tau were performed with NIH Image J software (version 1.34). The levels of pathological tau were normalized relative to β-actin.

#### Sandwich ELISA for phospho-tau (pS199) and (pS396)

Quantitative determination of human tau phosphorylated at pS396 and pS199 in 10% BH was performed using an Invitrogen Human tau ELISA kit (Invitrogen Corporation, Camarillo, CA) as we previously published [34]. Briefly, a monoclonal antibody specific for all forms of human tau (phosphorylated and non–phosphorylated) was pre-coated onto wells of microtiter strips. 10% BH were centrifuged at 20,000 × g for 20 min at 4°C. Samples diluted in the provided standard diluent buffer (1:200) were incubated for 2 hrs at room temperature allowing antigen to bind the immobilized (capture) antibody, followed by extensive washing and incubation for 1 hr at RT with detection antibody [rabbit polyclonal antibody specific for human tau (pS396 or pS199)] which binds to the immobilized human tau (pS396 or pS199) captured during the first incubation. After removal of excess antibody, horseradish peroxidase-labeled anti-rabbit IgG antibody was incubated for 30 min, followed by washing, and 3,3’,5,5’ -tetramethylbenzidine (TMB) substrate incubation to produce color. The intensity of this colored product was directly proportional to the concentration of human tau pS396 or pS199 present in the sample. The standards provided a linear curve and the best-fit line determined by linear regression were used to calculate human tau pS396 or pS199 concentration in our samples.

### Immune response analyses

Measurements of cytokine production to evaluate the Th subset induced and autoantibody responses towards Aβ42 were evaluated in plasma, utilizing the NYULMC Immune Monitoring Core. Animals were bled prior to immunization and every month thereafter including a sacrifice bleed. Since a limited amount of blood can be analyzed, the peripheral cytokine responses were determined in plasma at selected time points throughout the treatment period.

#### Cytokine assays

Cytokine profiles in plasma from CpG ODN-treated and control animals were analyzed using MILLIPLEX_MAP_ mouse cytokine/chemokine magnetic bead panel (EMD Millipore Corporation, Billerica, MA), after the i.p. injection of either CpG ODN or saline at 4 hrs post-injection every month throughout the course of treatment. T final (at the time of sacrifice) was collected 4 weeks after the last injection (after the completion of behavioral studies). Separate cytokine analyses were also performed in plasma samples collected 2 weeks after selected injections to evaluate the kinetic patterns of cytokine induction. Additionally, cytokine levels were assessed in single plasma samples collected from a separate cohort of 3xTg-AD mice during our short term study at the time of sacrifice (12 hrs post-injection). Brain cytokines levels were also screened 12 hrs after CpG ODN or saline i.p. administration. These assays allow quantification of an array of cytokines in a single small volume sample. A custom 10-plex detection kit, which measured IL1β, IL4, IL6, IL10, MCP1, TNFα, IFNγ, IL13, IP10 and IL12p70 was used. The manufacturer’s instructions were followed. Briefly, plasma samples (1:3 dilution - serum matrix) or supernatants of 10% (w/v) brain homogenates (centrifuged at 10,000 × g for 5 min at 4°C) were incubated with a mixture of Abs conjugated with fluorescent beads overnight. Following the biotinylated detection Ab and streptavidin-PE conjugate incubations, levels of each cytokine were measured using the Luminex 200™ analyzer (Immune Monitoring Core). Median Fluorescent Intensity (MFI) data were analyzed with ExPONENT software using a 5-parameter logistic curve-fitting method for calculating cytokine/chemokines concentrations in samples. Concentrations were calculated from standard curves and are expressed in pg/ml.

#### Aβ autoantibody response

The autoantibody levels were determined at 1:200 dilutions of plasma using ELISA as described previously in which 50 μg per plate of the Aβ42 peptide was coated onto microtiter wells (Immulon 2HB; Thermo, Waltham, MA) [9]. The antibodies in plasma were detected by a goat anti-mouse IgG linked to a horseradish peroxidase conjugate (Sigma; A8786) at 1:3000 dilution. Tetramethylbenzidine (TMB; Bio-Rad Laboratories Inc., Hercules, CA) was the development substrate.

### Statistical analysis

Data from the radial arm maze were analyzed by two-way repeated measures ANOVA followed by a Bonferroni’s *post hoc* test. Data from the accelerating rotarod and locomotor test were analyzed by one-way ANOVA. Differences between groups in total amyloid burden, fibrillar amyloid burden, tau burden, levels of extracted Aβ, levels of Aβ oligomers, phospho-tau/total tau levels in brain homogenates, Iba1, CD45, CD11b, CD206 activated microglia, GFAP astrogliosis, brain microhemorrhages, and cytokine levels were analyzed using a Student’s unpaired two-tailed *t* test or one-tailed *t* test. All statistical tests were performed using Prism 6.0 (Graphpad, San Diego, CA).

## Results

### Behavioral studies

**Figure 1:**
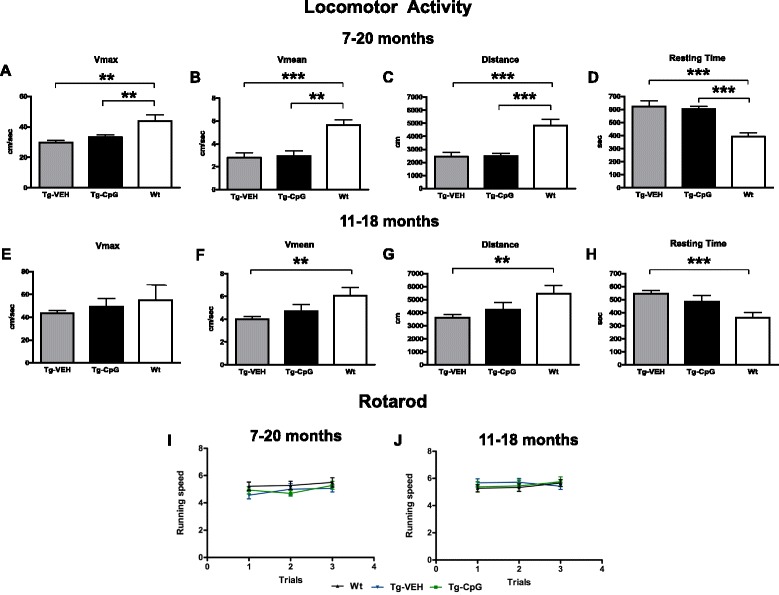
**Sensorimotor tests.** Locomotor activity test. **(** ***A*** **-** ***D*** **)** Treatment from 7–20 months. Both Tg groups were less active than their Wt age-matched littermates, but the CpG ODN-treated and control Tg mice did not differ significantly in any of 4 locomotor parameters measured [max speed **(** ***A*** **)**, average speed **(** ***B*** **)**, distance traveled **(** ***C*** **)**, resting time **(** ***D*** **)**]. **(** ***E*** **-** ***H*** **)** Treatment from 11–18 months. Tg mice were less active than Wt animals, but the maximum speed did not differ between the groups. **(** ***E*** **)** Our treated and control Tg groups did not differ in any of the locomotor parameters measured [max speed **(** ***E*** **)**, average speed **(** ***F*** **)**, distance traveled **(** ***G*** **)**, resting time **(** ***H*** **)**]. In addition, no significant differences were detected between CpG ODN Tg group and Wt mice. See Results section for *p* values. Rotarod. **(** ***I*** **-** ***J*** **)** No group differences were observed on the rotarod in both study groups. The error bars show SEM. This applies to all subsequent figures.

After treatment, at the age of 18 or 20 months, mice were subjected to behavioral testing. Behavioral analysis consisted of both a cognitive assessment and measurement of sensorimotor abilities. The latter tests were included to verify that performance on cognitive testing was not influenced by sensorimotor abnormalities. The transgenic (Tg) mice were less active than their wild-type (Wt) littermates, but significant differences were not observed between the treated and control Tg mice in any of the locomotor parameters measured (velocity, distance traveled, resting time) (Figure 1A-H). Group differences were observed in distance traveled (ANOVA, 7–20 months, *p* < 0.0001; 11–18 months, *p* = 0.03), average speed (ANOVA, 7–20 months, *p* = 0.001; 11–18 months, *p* = 0.009), resting time (ANOVA, 7–20 months, *p* < 0.0001; 11–18 months, *p* = 0.008) and maximum speed (ANOVA, 7–20 months, *p* = 0.0013; 11–18 months, *p* = 0.053).

Post hoc analysis revealed that the Wt mice traveled more distance, moved at a faster speed, and rested less than CpG ODN-treated Tg mice or Tg controls in the 7–20 month study group (maximum speed: Tg-CpG *vs* Tg-VEH, *p* = 0.19; Tg-CpG *vs* Wt, **p = 0.0068; Tg-VEH *vs* Wt, **p = 0.0026, Figure 1A; average speed: Tg-CpG *vs* Tg-VEH, *p* = 0.82; Tg-CpG *vs* Wt, **p < 0.002; Tg-VEH *vs* Wt, ***p = 0.0005, Figure 1B; distance traveled: Tg-CpG *vs* Tg-VEH, *p* = 0.86; Tg-CpG *vs* Wt, ***p < 0.0001; Tg-VEH *vs* Wt, ***p = 0.0008, Figure 1C; resting time: Tg-CpG *vs* Tg-VEH, *p* = 0.64; Tg-CpG *vs* Wt, ***p < 0.0001; Tg-VEH *vs* Wt, ***p = 0.0009, Figure 1D).

**Figure 2:**
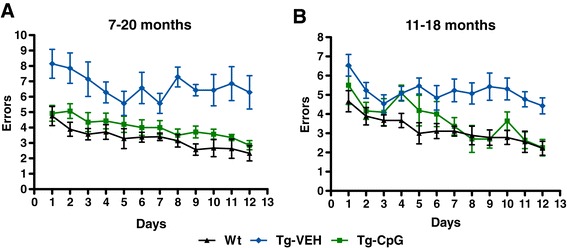
**Radial arm maze (working memory assessment).** TLR9 stimulation was effective at improving working memory in 3xTg-AD mice in both study groups. The CpG ODN-treated groups were better at navigating the maze than the vehicle-treated Tg mice. Significant differences were observed with CpG ODN-treated Tg mice performing comparably to their Wt littermates. **(** ***A*** **)** 7–20 months: Two-way repeated-measures ANOVA, group (treatment) effect, *p* < 0.0001; days effect, *p* < 0.0001; interaction (group *vs* days), *p* = 0.968. Bonferroni’s post hoc test revealed Tg-CpG *vs* Tg-VEH, *p* < 0.0001; Tg-CpG *vs* Wt, *p* = 0.16; Tg-VEH *vs* Wt, *p* < 0.0001. **(** ***B*** **)** 11–18 months: Two-way repeated-measures ANOVA, group (treatment) effect, *p* < 0.0001; days effect, *p* < 0.0001; interaction (group *vs* days), *p* = 0.795. Bonferroni’s post hoc test revealed Tg-CpG *vs* Tg-VEH, *p* < 0.001; Tg-CpG *vs* Wt, *p* = 0.2; Tg-VEH *vs* Wt, *p* < 0.001.

CpG ODN Tg mice and Tg controls in the 11–18 month study group performed in a similar matter in all of the measured locomotor parameters; however, there were significant differences between Wt and control Tg mice in three of the parameters (distance, resting time, average speed). The differences between Wt and CpG ODN Tg mice did not reach significance (maximum speed: Tg-CpG *vs* Tg-VEH, *p* = 0.39; Tg-CpG *vs* Wt, *p* = 0.69; Tg-VEH *vs* Wt, *p* = 0.26, Figure 1E; average speed: Tg-CpG *vs* Tg-VEH, *p* = 0.24; Tg-CpG *vs* Wt, *p* = 0.157; Tg-VEH *vs* Wt, **p = 0.003, Figure 1F; distance traveled: Tg-CpG *vs* Tg-VEH, *p* = 0.32; Tg-CpG *vs* Wt, *p* = 0.17; Tg-VEH *vs* Wt, **p = 0.003, Figure 1G; resting time: Tg-CpG *vs* Tg-VEH, *p* = 0.21; Tg-CpG *vs* Wt, *p* = 0.086; Tg-VEH *vs* Wt, ***p = 0.0003, Figure 1H). Maximum speed did not differ between the groups in the 11–18 month study group. No statistical differences were observed between the groups in their performance on the rotarod (Figure 1I-J). In addition to sensorimotor evaluation, the mice underwent a cognitive assessment. Working memory was evaluated using an eight-arm radial maze. As shown in Figures 2A and B, the overall performance (number of errors) of the mice differed significantly between Tg groups. CpG ODN-treated Tg groups navigated the radial arm maze with fewer errors than the vehicle-treated Tg groups, and their performance was similar to that of their age-matched Wt littermates. Vehicle Tg mice made significantly more errors than Wt animals [7 to 20 months: Two-way repeated-measures ANOVA, group (treatment) effect, *p* < 0.0001; days effect, *p* < 0.0001; interaction (group vs days), *p* = 0.968. Bonferroni’s *post hoc* test showed Tg-CpG *vs* Tg-VEH, *p* < 0.0001; Tg-CpG *vs* Wt, *p* = 0.16; Tg-VEH *vs* Wt, *p* < 0.0001; 11 to 18 months: Two-way repeated-measures ANOVA, group (treatment) effect, *p* < 0.0001; days effect, *p* < 0.0001; interaction (group *vs* days), *p* = 0.795. Bonferroni’s -*post hoc* test showed Tg-CpG *vs* Tg-VEH, *p* < 0.001; Tg-CpG *vs* Wt, *p* = 0.2; Tg-VEH *vs* Wt, *p* < 0.001]. The groups did not differ significantly in the time taken to run the maze (data not shown).

### Amyloid pathology

#### Quantification of amyloid burden

We next assessed the effects of CpG ODN in 3xTg-AD mice on Aβ pathology. The mice were sacrificed at 19 and 21 months of age after behavioral testing and the brains were processed for histology. Quantitative analysis was determined by stereological techniques, using an unbiased random sampling scheme and a semi-automated image analysis system as described previously [9].

**Figure 3:**
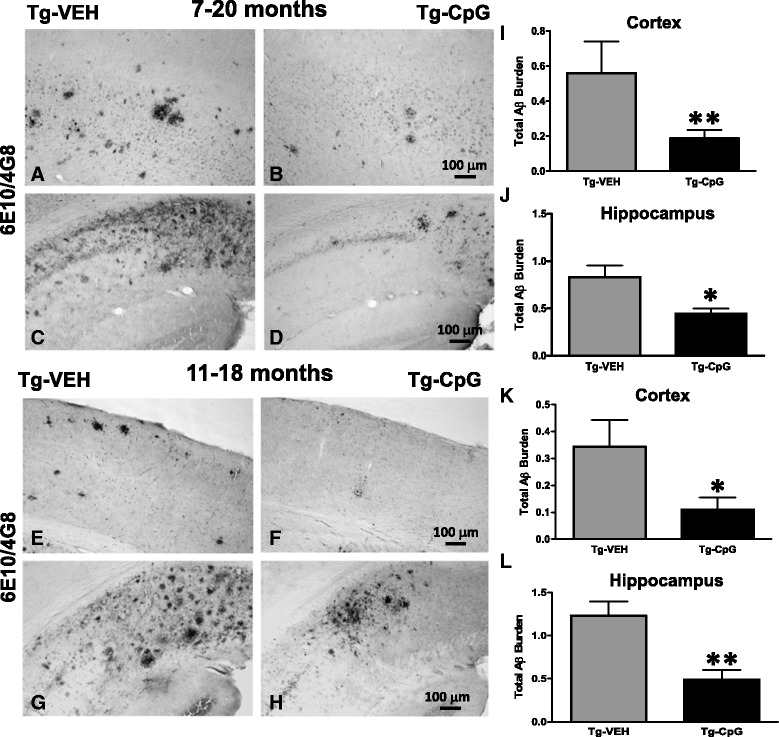
**Total amyloid burden.** Immunohistochemistry (6E10/4G8) showed a notable reduction in total amyloid burden in cortical and hippocampal sections of CpG ODN-treated mice compared with sections of vehicle-treated mice in both study groups **(** ***A*** **-** ***H*** **)**. Quantitative stereological analysis of total amyloid burden revealed a significant reduction in 3xTg-AD mice treated with CpG ODN compared with control Tg mice. There was a 67% reduction (**p = 0.018) in total cortical amyloid burden **(** ***I*** **)** and 47% reduction (*p = 0.0032) in total hippocampal amyloid burden **(** ***J*** **)** in the 7–20 month study group. Similarly, there was a 64% reduction (*p = 0.047) in total cortical amyloid burden **(** ***K*** **)** and 60% reduction (**p = 0.0013) in total hippocampal amyloid burden **(** ***L*** **)** in the 11–18 month study group. Scale bars (100 μm) in ***B*** and ***F*** correspond to cortical images ***A***, ***B***, ***E***, ***F***. Scale bars in ***D*** and ***H*** correspond to hippocampal images ***C***, ***D***, ***G***, ***H***.

**Figure 4:**
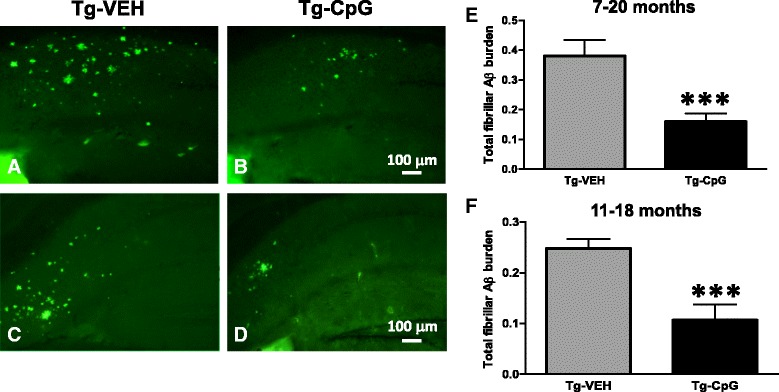
**Fibrillar amyloid burden.** Thioflavine-S staining revealed significant differences in hippocampal fibrillar amyloid burden between CpG ODN-treated and vehicle-treated 3xTg-AD mice **(** ***A*** **-** ***D*** **)**. Thioflavine-S staining of amyloid depositions was minimal in the cortex. There was a 58% reduction (***p = 0.0004) in hippocampal fibrillar amyloid burden in the 7–20 month study group **(** ***E*** **)** and a 57% reduction (***p = 0.0004) in hippocampal fibrillar amyloid burden in the 11–18 month study group **(** ***F*** **)** as quantified using an unbiased random sampling scheme and semi- automated image analysis system. Scale bar, 100 μm.

**Figure 5:**
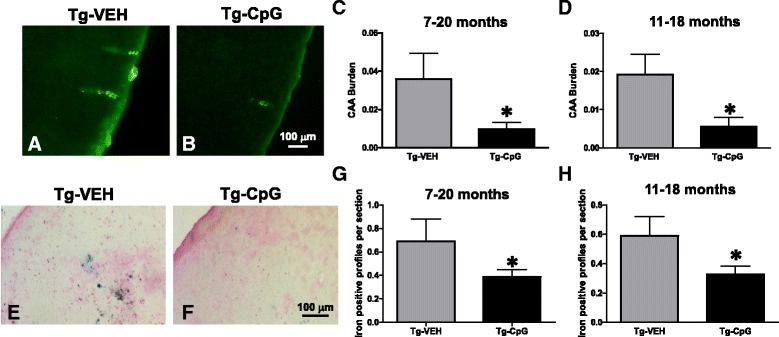
**Vascular amyloid (CAA burden) and brain microhemorrhages.** Representative images of a visible reduction in the CAA burden of the cortical vessels in mice treated from 7–20 months are shown in ***A*** and ***B***. Significantly, CpG ODN treatment led to a 74% (*p = 0.03) reduction in the vascular amyloid burden in the 7–20 month study group **(** ***C*** **)** and an 80% reduction (*p = 0.036) was noted in CpG ODN-treated animals in the 11–18 month study group **(** ***D*** **)** measured by unbiased stereology. ***E*** and ***F*** show representative 3xTg-AD mice brain sections stained with Perl’s stain for ferric iron in haemosiderin. Quantification of CAA-associated microhemorrhages revealed a significant reduction of iron positive profiles per brain section in CpG ODN-treated animals in both study groups (7–20 study group, *p = 0.034; 11–18 study group, *p = 0.043) **(** ***G*** **,** ***H*** **)**. Scale bar, 100 μm.

Histological observation in 3xTg-AD mice indicated that CpG ODN-treated mice have fewer plaques compared to vehicle Tg mice as visualized by immunostaining (using anti-Aβ 6E10/4G8) in both study groups (Figure 3A-H). In the 7–20 month study group, peripheral administration of TLR9 agonist CpG ODN led to a 67% reduction (two-tailed *t* test, **p = 0.018) in total cortical amyloid burden and a 47% reduction (*p = 0.0032) in total hippocampal amyloid burden compared to age-matched vehicle Tg animals (Figure 3I-J). In the 11–18 month study group, there was a 64% reduction (*p = 0.047) in total cortical amyloid burden and a 60% reduction (**p = 0.0013) in total hippocampal amyloid burden in 3xTg-AD mice treated with CpG ODN compared to control Tg mice treated with vehicle (Figure 3K-L). Thioflavine-S staining was used to demonstrate fibrillar amyloid plaque accumulation and CAA load (Figure 4A-D). Quantitative assessment of hippocampal fibrillar amyloid burden revealed a significant 58% reduction (two-tailed *t* test, ***p = 0.0004) in CpG ODN-treated animals compared to controls in the 7–20 month study group (Figure 4E). A 57% reduction (***p = 0.0004) of the fibrillar amyloid burden was observed in the 11–18 month study group (Figure 4F). Thioflavine-S staining of amyloid depositions was observed mainly in the hippocampus. Moreover, visible reductions were revealed in the CAA burden of the penetrating cortical and hippocampal vessels in the CpG ODN-treated animals compared to controls in both age groups (Figure 5A-B). CpG ODN treatment led to a 74% reduction (two-tailed *t* test, *p = 0.03) in the vascular amyloid burden in the 7–20 study group (Figure 5C). Similar effects were seen in the 11–18 month study group. An 80% reduction (*p = 0.036) in the vascular amyloid was noted in the CpG ODN-treated animals (Figure 5D). Brain microhemorrhages were detected in 3xTg mouse brain sections stained with Perl’s stain. Following treatment with CpG ODN we observed a significant decrease in the extent of cerebral microhemmorhages (7–20 study group, one-tailed *t* test, *p = 0.034; 11–18 study group, one-tailed *t* test, *p = 0.043) (Figure 5E-H).

#### Assessment of Aβ levels, Aβ oligomers in the brain

**Figure 6:**
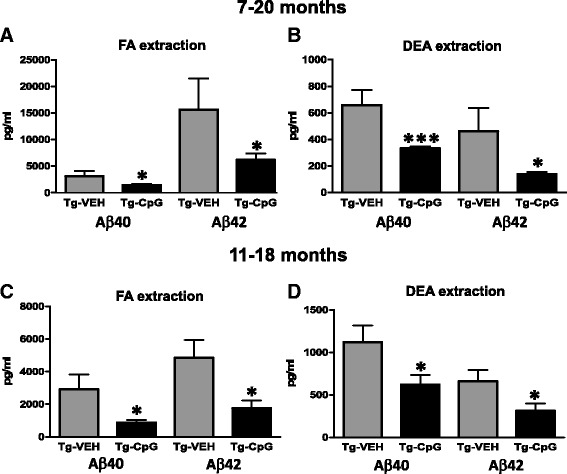
**Brain Aβ levels.** Treatment from 7–20 months significantly lowered total (FA extracted) Aβ40 and Aβ42 levels by 54% (*p = 0.04) and 61% (*p = 0.04), respectively **(** ***A*** **)**. Soluble (DEA extracted) Aβ40 and Aβ42 fractions were also significantly reduced by 50% (***p = 0.0009) and 70% (*p = 0.019), respectively **(** ***B*** **)**. Treatment in the 11–18 month study group significantly decreased total Aβ40 and Aβ42 brain levels by 69% (*p = 0.03) and 64% (*p = 0.026), respectively **(** ***C*** **)** and soluble Aβ40 and Aβ42 by 44% (*p = 0.047) and 53% (*p = 0.019) **(** ***D*** **)**, as assessed by sandwich ELISA.

Stimulation of TLR9 signaling significantly decreased total (FA extract) and soluble (DEA extract) brain Aβ levels in 3xTg-AD mice, in both study groups. ELISA measurements revealed a statistically significant decrease in the levels of total (FA extracted) Aβ40 and Aβ42 by 54% (two tailed *t* test, *p = 0.04) and 61% (*p = 0.04), respectively, after the CpG ODN treatment in the 7–20 month study group (Figure 6A). The levels of soluble (DEA extracted) Aβ40 and Aβ42 fractions were significantly reduced by 50% (two-tailed *t* test, ***p = 0.0009) and 70% (*p = 0.019), respectively, in CpG ODN-treated mice in the 7–20 month study group (Figure 6B). Treatment from 11 to 18 months significantly lowered total Aβ40 and Aβ42 by 69% (one-tailed *t* test, *p = 0.03) and 64% (two-tailed *t* test, *p = 0.026), respectively, and soluble Aβ40 and Aβ42 brain levels by 44% (two-tailed *t* test, *p = 0.047 and 53% *p = 0.019) (Figure 6C-D).

**Figure 7:**
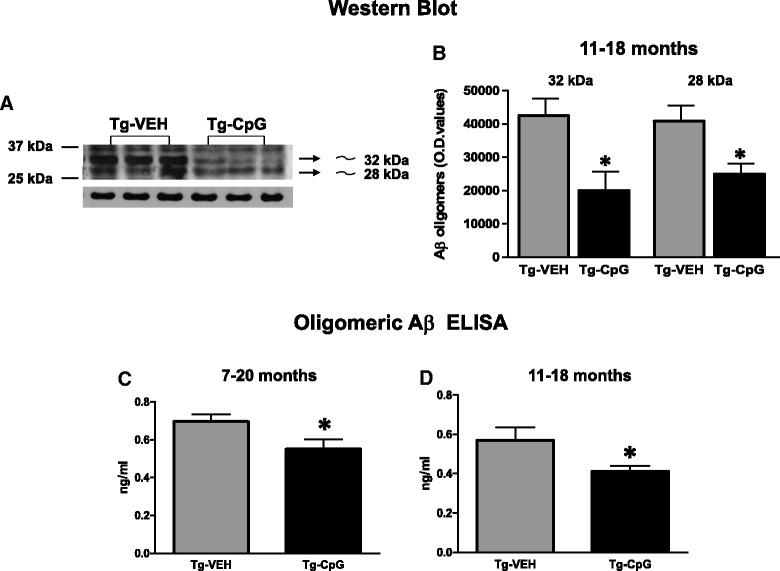
**Assessment of Aβ oligomers.** Western blot of brain homogenates labeled with A11 oligomer-specific antibody **(** ***A*** **)** and densitometric analysis of A11 immunoreactive oligomer specific bands (approximately 32 kDA and 28 kDa, in arbitrary O.D. units) showed a significant reduction in CpG ODN-treated Tg mice compared with control Tg mice in the 11–18 month study group (32 kDA, *p = 0.015; 28 kDa, *p = 0.023) **(** ***B*** **)**. As further assessed by an ELISA specific to oligomeric Aβ, CpG ODN treatment led to a significant decrease in Aβ oligomers in both study groups (7–20 month study group, *p = 0.03; 11–18 study group, *p = 0.04) **(** ***C*** **,** ***D*** **)**.

Soluble Aβ oligomers (also known as ADDLs) may account for synaptotoxicity and cognitive impairment, thus presenting a significant therapeutic target. Pathogenic Aβ oligomers in the brain homogenates were assessed by Western blot using the A11 oligomer-specific antibody (Figure 7A-B). CpG ODN treatment led to a significant decrease in the level of A11 immunoreactive (approximately 32 kDA and 28 kDa) oligomers in the 11–18 month study group (32 kDA, two-tailed *t* test, *p = 0.015; 28 kDa, two-tailed *t* test, *p = 0.023). No difference was found in the 7–20 month study group (data not shown). We focused on oligomers in the range of ~32 kDa similar to our prior published work and that of others [30,35,36]. Different groups have focused on oligomers in the ~56 kDa range [37]. However, it has been demonstrated that Aβ oligomers can, in part, become dissociated during processing and when run on SDS-polyacrylamide gel electrophoresis [38], leading to variation in their molecular weight.

Due in part to this variability, we further evaluated CpG ODN treatment effect by an ELISA specific to oligomeric Aβ as previously published [18,19]. Stimulation of TLR9 signaling significantly decreased Aβ aggregates/oligomers in 3xTg-AD mice, in both study groups (7–20 month study group: one-tailed *t* tes*t*, *p = 0.03; 11–18 month study group: two-tailed *t* tes*t*, *p = 0.04, Figure 7C-D).

### Tau pathology

#### Quantification of Tau burden

**Figure 8:**
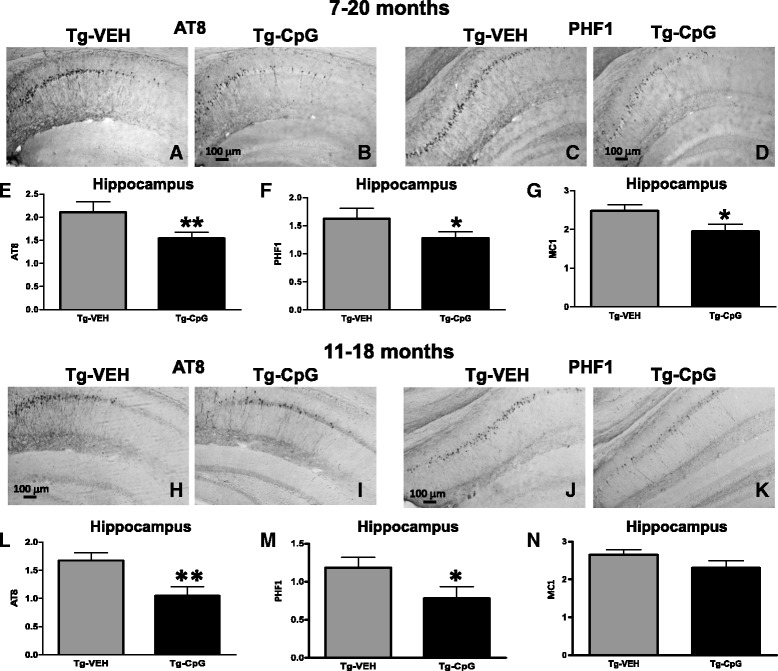
**Histological evaluation of pathological tau.** Representative images of AT8 and PHF1 immunostaining within the hippocampus depicted differences between CpG ODN-treated and vehicle-treated 3xTg-AD mice in both study groups **(** ***A*** **-** ***D*** **)**, **(** ***H*** **-** ***K*** **)**. Semiquantitative analysis of hippocampal neurons revealed reductions in AT8 (**p = 0.028) **(** ***E*** **)**, PHF1 (*p = 0.049) **(** ***F*** **)** and MCI (*p = 0.036) **(** ***G*** **)** immunoreactivity in treated mice compared with control mice in the 7–20 month study group. Likewise, significant reductions were observed in AT8 (**p = 0.0075) **(** ***L*** **)** and PHF1 (*p = 0.03) **(** ***M*** **)** immunoreactive tau in CpG ODN-treated animals in the 11–18 month study group. There was a trend for reduced MCI immunoreactivity in treated animals from 11 to 18 months (*p* = 0.06) **(** ***N*** **)**. Scale bars, 100 μm.

To determine the efficacy of CpG ODN on the tau pathology we immunohistochemically evaluated sections from CpG ODN- and vehicle-treated 3xTg-AD mice with different anti-tau antibodies. The early conformation specific antibody MC1, and antibodies recognizing phospho-tau epitopes Ser202/Thr205 (AT8) and Ser396/Ser404 (PHF1) are among the most characterized identifiers of neurofibrillary pathology. The effects were mainly limited to the hippocampus since cortical MC1, AT8 and PHF1 immunoreactivity was minimal. Significant reductions of phospho-tau were noted in CpG ODN-treated animals in both study groups. Semiquantitative analysis of neuronal immunoreactivity in the hippocampus revealed significant reductions in AT8 (two-tailed *t* test, **p = 0.028) PHF1 (one-tailed *t* test, *p = 0.049) and MC1 (one-tailed *t* test, *p = 0.036) tau markers in CpG ODN-treated 3xTg-AD mice compared with control mice that received vehicle only in the 7–20 month study group (Figure 8E-G).

**Figure 9:**
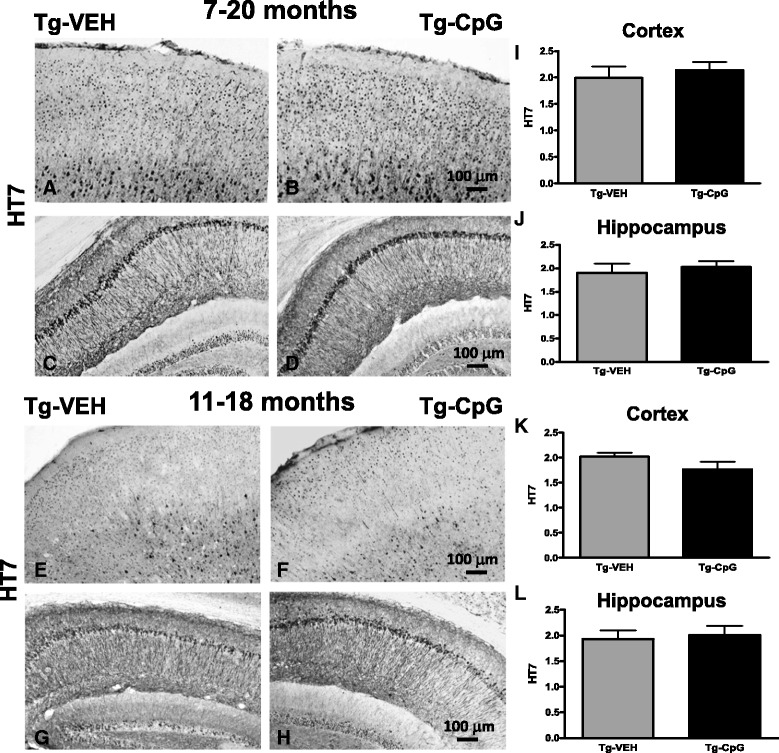
**Histological evaluation of total tau.** Representative images of HT7 immunostained cortical and hippocampal brain sections from control and treated mice **(** ***A*** **-** ***H*** **)**, and subsequent semiquantitative analysis **(** ***I*** **-** ***L*** **)** did not reveal any significant differences in total tau levels between CpG ODN-treated and vehicle-treated Tg animals in both study groups. Scale bars (100 μm) in ***B*** and ***F*** correspond to cortical images ***A***, ***B***, ***E***, ***F***. Scale bars in ***D*** and ***H*** correspond to hippocampal images ***C***, ***D***, ***G***, ***H***.

AT8 and PHF1 immunostained coronal sections of mice treated from 11 to 18 months indicated visible differences between the groups (Figure 8H-K). There were significant reductions in AT8 (two-tailed *t* test, **p = 0.0075) and PHF1 (one-tailed, *p = 0.03) immunoreactivity in CpG ODN-treated 3xTg-AD mice compared with vehicle-treated Tg controls. Strong trend for a diminished MC1 (one-tailed *t* test, *p* = 0.06) immunoreactivity was observed in treated animals compared with controls in the 11–18 month study group (Figure 8L-N). In addition, the level of total tau (HT7 immunoreactivity) was unaffected by the treatment in both study groups (Figure 9).

#### Assessment of phosphorylated and total tau in the brain

**Figure 10:**
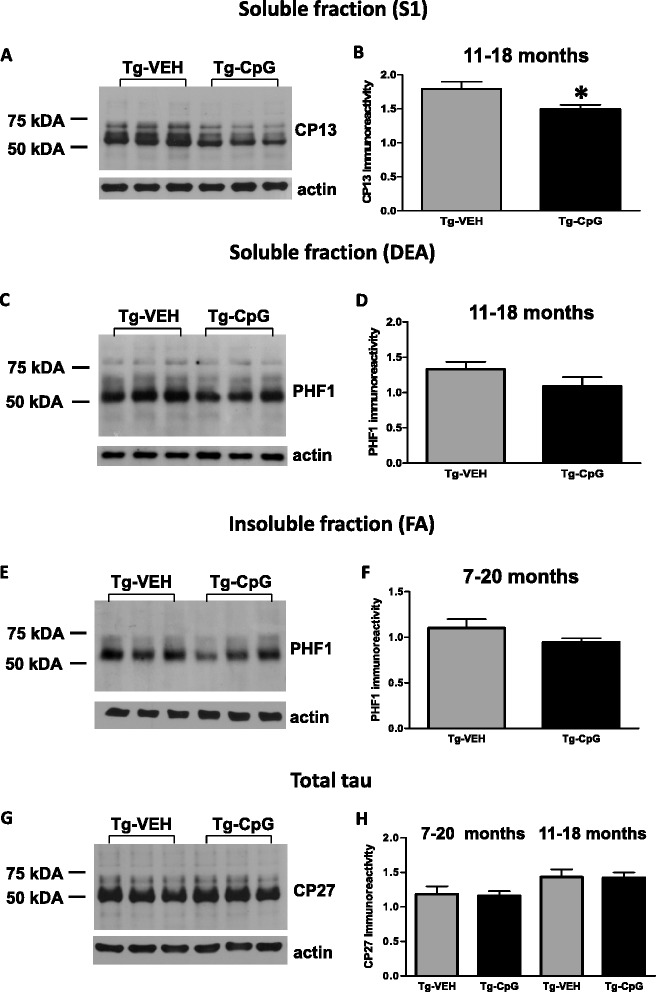
**Western blot analysis of phosphorylated and total tau.** Densitometric analysis showed a significant reduction in soluble (S1 fraction) CP13 phospho-tau levels (*p = 0.035) when normalized to actin, in the 11–18 month study group **(** ***A*** **,** ***B*** **)**. Additional analysis indicated a trend for a decrease in PHF1 reactive tau in the same treatment group (*p* = 0.07) (data not shown). No statistically significant changes were observed in Western blot analysis of DEA soluble brain fraction, but there was a notable trend in reduction of PHF1 (*p* = 0.086) **(** ***C*** **,** ***D*** **)** and CP13 DEA soluble tau levels (data not shown) in CpG ODN-treated mice in the 11–18 month study group. The groups did not differ significantly in their levels of insoluble (FA brain fraction) CP13 or PHF1 tau; however, there was a trend for reduced insoluble PHF1 tau levels in the CpG ODN-treated animals compared to controls in the 7–20 month study group (*p* = 0.08) **(** ***E*** **,** ***F*** **)**. Furthermore, no differences in total tau assessed by CP27 were detected between our treated and control 3xTg-AD animals. Total tau levels were not affected by CpG ODN treatment in both study groups **(** ***G*** **,** ***H*** **)**. ***G*** shows representative western blot from control and CpG ODN mice treated from 7 to 20 months. The same amount of protein was loaded in each lane of the Western blots.

To further determine the extent of tau pathology in 3xTg-AD mice after CpG ODN administration, we carried out multiple biochemical analyses. Standard Western blot analysis was first performed on soluble brain extracts that represents the supernatant obtained after centrifuging the brain homogenate at low speed (S1). Total tau was measured with TG5 (total human and mouse tau) and CP27 (total human tau) antibody whereas pathological tau was detected with phosphorylation site-specific monoclonal antibodies PHF1 (Ser396/Ser404) and CP13 (Ser202). Densitometric analysis revealed significant reduction in soluble CP13 tau levels (two tailed *t* test, *p = 0.035), when normalized to actin, after CpG ODN treatment in the 11–18 month study group (Figure 10A-B). A strong trend was observed for a decrease in PHF1 reactive tau (one tailed *t* test, *p* = 0.07, data not shown). No differences were found on PHF1 or CP13 tau blots in the 7–20 month study group (data not shown). To further determine the levels of soluble and insoluble tau we performed sequential biochemical extractions. Measurements of soluble tau in DEA brain fraction did not reveal a reduction between the groups in the 7–20 month study group. However, there was a strong trend toward reduced PHF1 (one tailed *t* test, *p* = 0.086, Figure 10C-D) and CP13 tau levels (data not shown) in the DEA soluble fraction for CpG ODN-treated animals in the 11–18 month study group. Insoluble tau protein was extracted with FA. Our further analysis in FA brain fraction indicated a trend for reduced insoluble PHF1 tau levels in the CpG ODN-treated animals compared to controls in the 7–20 month study group (one tailed *t* test, *p* = 0.08, Figure 10E-F). No statistical differences in the levels of PHF1 or CP13 immunoreactivity were detected between CpG ODN-treated mice and control animals in the 11–18 month study group (data not shown). Furthermore, total tau levels (CP27) (Figure 10G-H) and TG5 (data not shown) remained unchanged in all treatment groups.

**Figure 11:**
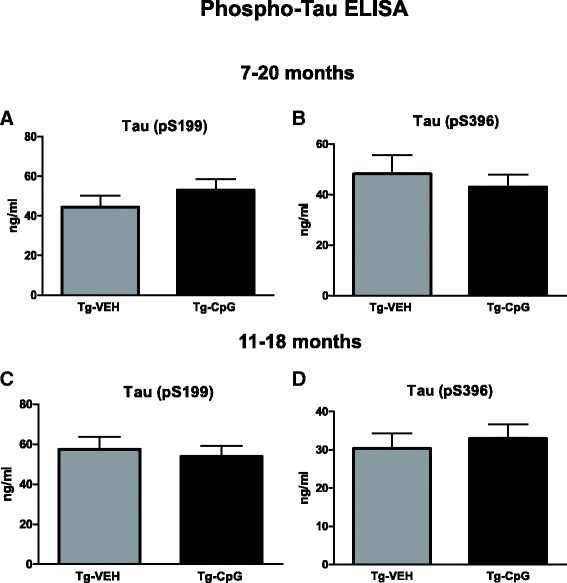
**Assessment of phosphorylated and total tau by ELISA.** Further quantitative analysis of phospho-tau (pS396) and (pS199) was performed in brain homogenate fractions of the whole hemisphere by using ELISA and showed no differences between our treated and control animals in both study groups **(** ***A*** **-** ***D*** **)**.

A growing number of studies suggest that altered inflammation in the brain can influence subsequent development of tau pathology [11-16]. To confirm that stimulation of innate immunity with TLR9 agonist, CpG ODN, does not accelerate formation of tau depositions as previously demonstrated in a number of studies using TLR4 agonists (LPS) [16,39,40], we performed additional analyses of tau pathology. Quantitative determination of tau phosphorylated at (pS396) and (pS199) in 10% BH was performed by ELISA using a Human Tau ELISA kit, and showed no differences between the groups (Figure 11A-D). Overall, our thorough biochemical analyses did not show as robust treatment effect as observed with immunohistochemistry. This may be related to the fact that all biochemical analyses were performed in brain fractions of the whole hemisphere and not from fractions of micro-dissected specific brain regions.

### Associated histopathology

#### Assessment of microgliosis and astrogliosis

**Figure 12:**
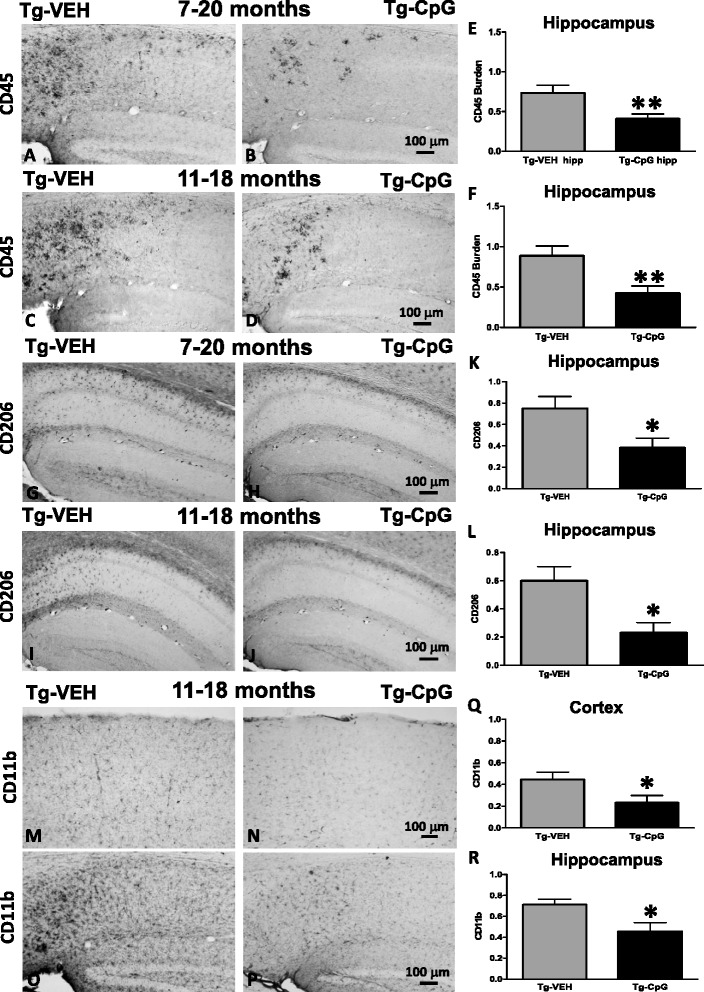
**Assessment of CD45, CD206 and CD11b microgliosis.** CpG ODN reduced overall hippocampal CD45 immunoreactivity in both treatment groups **(** ***A*** **-** ***D*** **)**. Quantitative stereological analysis revealed 44% reduction (**p = 0.008) in CD45 reactive microglia burden in the 7–20 month study group and 52% reduction (**p = 0.0065) in the 11–18 month study group **(** ***E*** **,** ***F*** **)**. Cortical CD45 immunoreactivity was minimal. Scale bar, 100 μm. Immunostaining with CD206 **(** ***G*** **-** ***J*** **)** followed by semiquantitative analysis demonstrated significant reduction in hippocampal CD206 positive cells in both age groups (7–20 months, *p = 0.04; 11–18 months, *p = 0.013) **(** ***K*** **,** ***L*** **)**. Cortical CD206 was minimal. The degree of CD206 microgliosis was graded on a scale of 0–4. Scale bar, 100 μm. Representative immunostained images with CD11b microglia marker **(** ***M*** **-** ***P*** **)** and subsequent semiquantitative analysis of CD11b immunoreactivity revealed marked cortical (*p = 0.037) and hippocampal (*p = 0.015) reductions in CpG ODN-treated mice in the 11–18 month study group **(** ***Q*** **,** ***R*** **)**. The degree of CD11b microgliosis was analyzed on a scale of 0–4. Scale bar (100 μm) in ***N*** corresponds to cortical images ***M***, ***N***. The scale bar in ***P*** corresponds to hippocampal images ***O***, ***P***.

**Figure 13:**
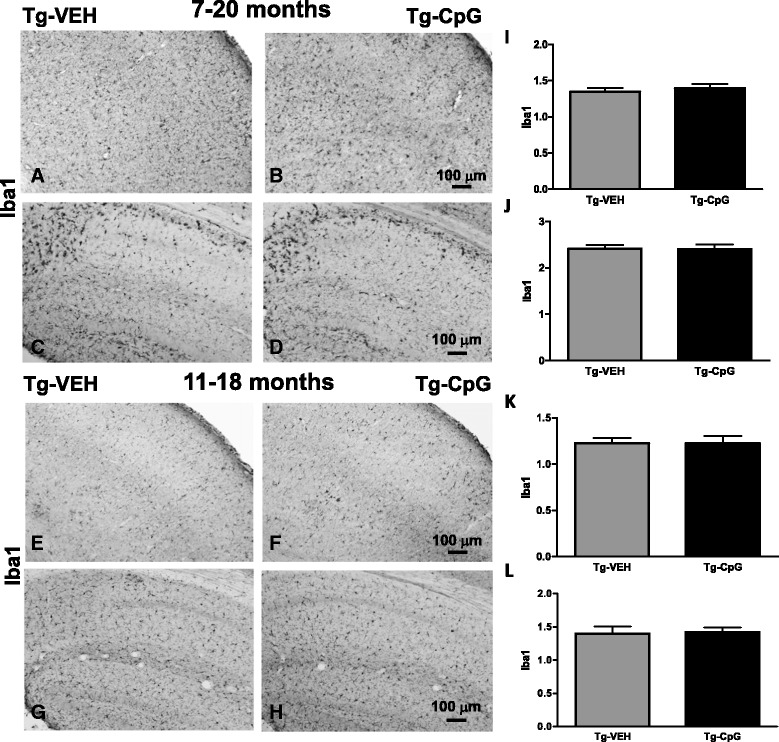
**Assessment of Iba1 microgliosis.** Histological observations **(** ***A*** **-** ***H*** **)** and semiquantitative rating of Iba1 microglial staining did not reveal group differences in the degree of cortical and hippocampal Iba1 immunoreactivity **(** ***I*** **-** ***L*** **)**. Iba1 microgliosis was rated on a scale of 0–4. Scale bars (100 μm) in ***B*** and ***F*** correspond to cortical images ***A***, ***B***, ***E***, ***F***. Scale bars in ***D*** and ***H*** correspond to hippocampal images ***C***, ***D***, ***G*** ,***H***.

Subsequent evaluation of CpG ODN treatment effect on microglial activation was performed. Analysis of commonly used microglial and macrophage marker CD45, which is typically expressed in association with more mature plaques [23], indicated significant differences between groups. An overall hippocampal reduction in CD45 microglial activity in CpG ODN- treated mice was observed at the end of the study in both age groups (Figure 12A-D). Quantitative stereological analysis demonstrated 44% reduction (two-tailed *t* test, **p = 0.008) in CD45 reactive microglia burden in the 7–20 month study group and 52% reduction (**p = 0.0065) in the 11–18 month study group (Figure 12E-F). The staining intensity of CD45 marker was very low in the cortex (data not shown). Similarly, 3xTg-AD mice treated with CpG ODN exhibited an overall decrease in hippocampal CD206 (marker of M2 state of alternative activation) immunoreactivity at the end of the treatment (two-tailed *t* test, 7–20 months, *p = 0.04; 11–18 months, *p = 0.013, Figure 12G-L). Cortical CD206 immunoreactivity was minimal and was not quantitated (data not shown). The assessment of another well-established microglial/mononuclear phagocyte marker CD11b was based on semiquantitative analysis of the extent of microgliosis. CpG ODN reduced overall cortical (two-tailed *t* test, *p = 0.037) and hippocampal (*p = 0.015) CD11b immunoreactivity in the 11–18 month study group (Figure 12M-R). No difference in CD11b reactive microglia was found in the 7–20 month study group (data not shown). The Iba1 microglial marker labels both resting and activated microglia populations [28,29]. In contrast, no differences in cortical and hippocampal Iba1 immunoreactivity were found comparing CpG ODN-treated and vehicle-treated animals in both study groups (Figure 13). As assessed by GFAP immunoreactivity, followed by semiquantitative analysis, no significant differences were observed between the groups in the degree of astrogliosis (data not shown). To further determine if any neuroinflammation was induced, the brains were examined for the presence of lymphocytic infiltration. No T cells (CD3) were detected in any region of the brain in treated and control mice (data not shown). Hence, there was no evidence of cerebral inflammatory toxicity in the brains of CpG ODN- treated mice.

**Figure 14:**
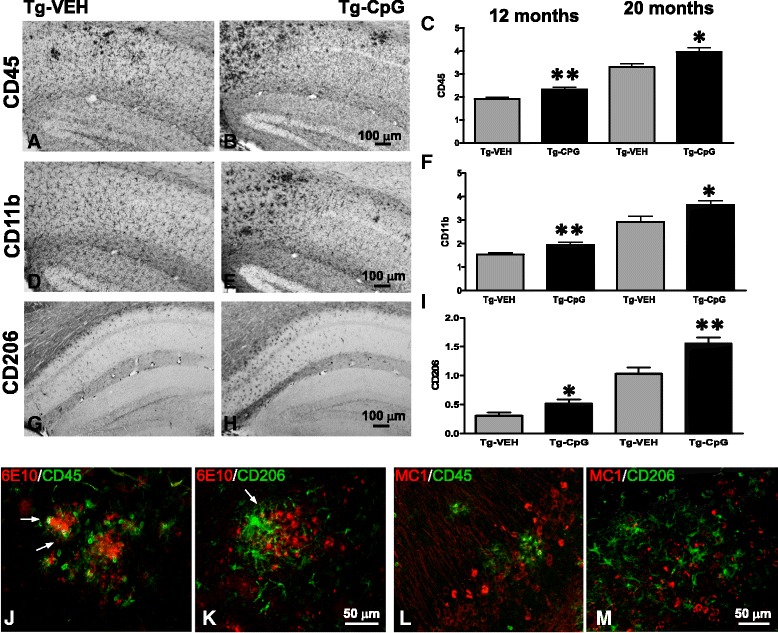
**Assessment of CD45, CD11b and CD206 microglial activation states 12 hrs post CpG ODN injection.** An acute injection of CpG ODN led to an induction of favorable microglia/macrophage activation in 3xTg-AD mice. Immunohistochemistry indicated a significant increase in CD45 (young, **p = 0.001; aged, *p = 0.01) **(** ***A*** **,** ***B*** **)**, CD11b (young, **p = 0.0013; aged, *p = 0.02) **(** ***D*** **,** ***E*** **)** and CD206 (young, *p = 0.017; aged, **p = 0.006) **(** ***G*** **,** ***H*** **)** macrophage/microglia markers 12 hrs after CpG ODN administration in both age cohorts of 3xTg-AD mice. The assessment of microglial cell activation after a single i.p. injection of either CpG ODN or saline was based on semiquantitative analysis of the extent of microgliosis in the hippocampus **(** ***C*** **,** ***F*** **,** ***I*** **)**. Scale bars, 100 μm. CD45 and CD206 positive cells, likely to be infiltrating peripheral macrophages, were clearly restricted to AD pathology. Co-staining of microglial markers (CD45 or CD206, green) and Aβ deposits (6E10/4G8, red) revealed an increase in CD45 and CD206 immunoreactive microglia around the plaques in mice injected with CpG ODN, 12 hrs post CpG ODN injection. Confocal microscopy confirmed partial co-localization of CD45 and CD206 positive microglia with 6E10/4G8 positive Aβ deposits **(** ***J*** **,** ***K*** **)**. Arrows indicate co-localization. In addition, double immunofluorescence for microglial markers (CD45 or CD206, green) and pathological tau (MC1, red) indicated an increase in CD45 and CD206 microglial activation in association with MC1 reactive pathological tau within hippocampal neuronal perikarya and neuronal processes, as well as dystrophic neurites **(** ***L*** **,** ***M*** **)**. ***J*** - ***M*** show representative hippocampal images from 20 month old Tg-control and Tg-CpG ODN mice. Similar increase in activation of CD45 and CD206 microglial cells in the vicinity of AD pathology was observed in 12 month old CpG ODN-injected animals (data not shown). Scale bars, 50 μm.

**Figure 15:**
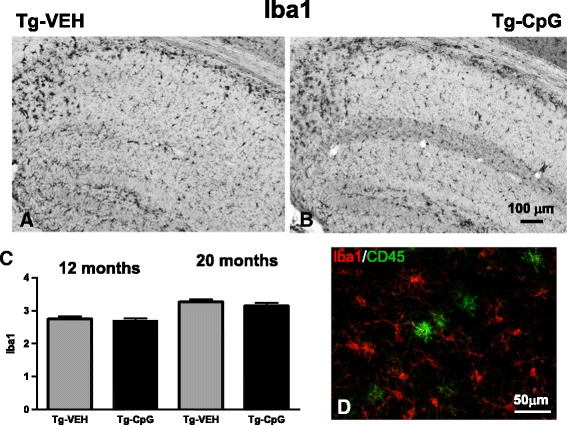
**Assessment of Iba1 microglial activation 12 hrs post CpG ODN injection.** Accumulation of Iba1 immunoreactive cells was not affected by acute injection of CpG ODN. Representative hippocampal images **(** ***A*** **,** ***B*** **)** and subsequent semiquantitative comparisons of Iba1 reactive microglia showed no group differences in both age cohorts **(** ***C*** **)**. Scale bar, 100 μm. Note that double immunofluorescence labeling with CD45 (green) and Iba1 (red) did not reveal co-localization **(** ***D*** **)**. Scale bar, 50 μm.

An acute administration of CpG ODN in a separate cohort of 3xTg-AD mice was performed to further evaluate the effects of TLR9 signaling on the macrophage/microglia activation state and ability to promote phagocytosis. Function and expression of TLRs can be affected by immunosenescence in old animals; hence our short term experiment was performed in young (12 months old) and aged (20 months old) animals. Semiquantitative analysis showed a significant increase in hippocampal CD45 (two-tailed *t* test, young, **p = 0.001; aged, *p = 0.01, Figure 14A-C) and CD11b (two-tailed *t* test, young, **p = 0.0013; aged, *p = 0.02, Figure 14D-F) microglial markers in the brains of 3xTg-AD animals 12 hrs after CpG ODN i.p. administration in both age groups. No significant differences were noted in microglial density in the cortex (data not shown). Additional staining was conducted to further analyze the microglial phenotype using CD206, an alternative M2 macrophage activation marker [24,26,27]. When examining the stained sections, we noted an increased CD206 hippocampal microglial activation in the CpG ODN injected animals (two-tailed *t* test, young, *p = 0.017; aged, **p = 0.006, Figure 14G-I). The cortical staining intensity of CD206 was minimal (data not shown). Moreover, CD45 and CD206 immunostaining counterstained with anti-Aβ 6E10/4G8 further confirmed the acute activation state of microglia in close proximity to Aβ deposits, by revealing an increase in activated microglia around the plaques in mice injected with CpG ODN compared to controls (saline injection) in both age groups (Figure 14J-K). Partial co-localization of CD45 or CD206 positive microglia with 6E10/4G8 positive Aβ deposits was often observed in the CpG ODN groups (Figure 14J-K). Subsequently, we co-stained the brain sections with pathological tau marker (MC1). Double immunofluorescence for MC1 reactive pathological tau and both microglial markers (CD45 and CD206) demonstrated dense gathering of activated microglia around pathological tau within hippocampal neuronal perikarya and processes, as well as dystrophic neurites in CpG ODN-treated mice in both age cohorts (Figure 14L-M). No co-localization was noted between microglial markers and MC1 immunoreactivity. The microglia/macrophage activation was increased in the subiculum and CA1 regions of 3xTg-AD mice, which corresponded to the regions of the most intense amyloid and tau depositions. On the other hand, we found that administration of CpG ODN did not alter Iba1 immunoreactivity. No differences were detected in the number of Iba1 positive cells between groups (Figure 15A-C). Furthermore, co-staining for Iba1 and CD45 microglia did not reveal co-localization (Figure 15D). These observations highlight the importance of assessing the activation states of microglia that may have contributed to CpG ODN therapeutic outcomes.

### Characterization of immune responses

#### Aβ autoantibody levels

**Figure 16:**
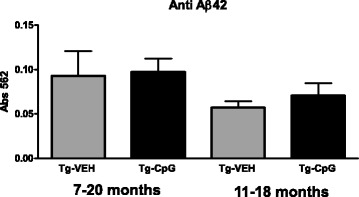
**Levels of autoantibodies.** No apparent differences were observed in the autoantibody response towards Aβ42 in plasma collected at the end of the study among CpG ODN-treated and vehicle-treated Tg mice in both study groups, as assessed by ELISA.

We next set out to test whether the CpG ODN therapeutic effect had any relationship with the production of anti-Aβ antibodies. The autoantibody response towards Aβ42 was assessed periodically. Stimulation of TLR9 signaling did not lead to generations of anti-Aβ42 antibody in CpG ODN-treated Tg mice when compared with vehicle-treated Tg mice. No differences were observed in the levels of autoantibodies in plasma obtained at the end of the study in both age groups (Figure 16). Hence, stimulation of innate immunity with CpG ODN did not lead to secondary activation of adaptive immunity against Aβ.

#### Cytokine responses (plasma)

**Figure 17:**
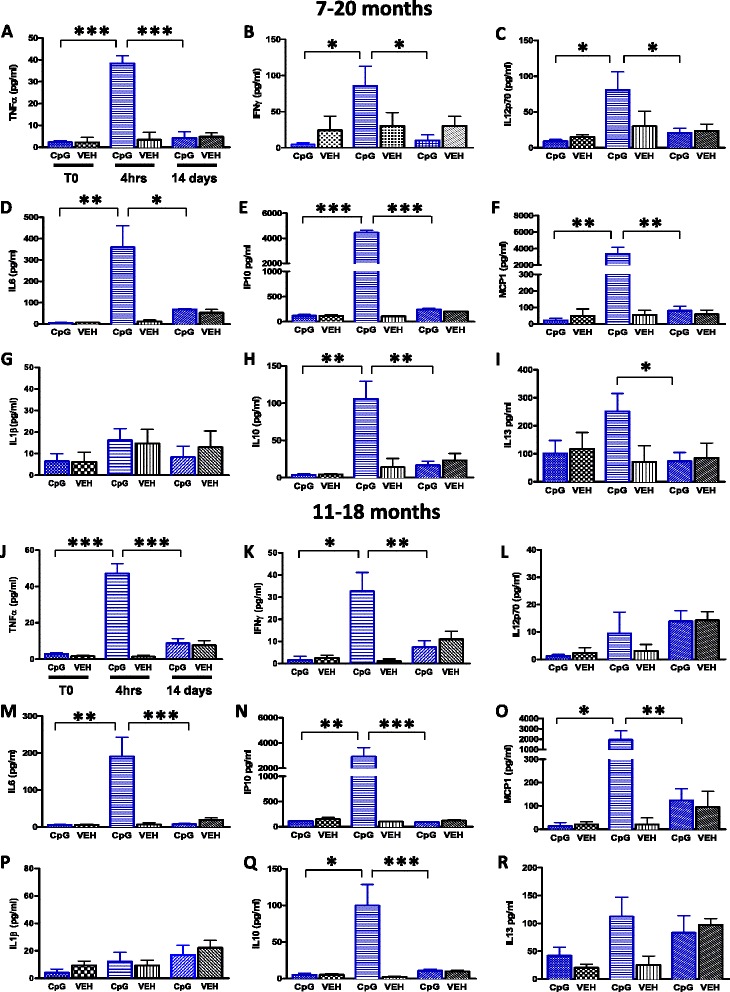
**Plasma cytokine/chemokine analysis (long term study).** Luminex analysis (Th1/Th2 mouse cytokine/chemokine detection kit) was used to determine immunostimulatory responses induced by CpG ODN in the plasma of 3xTg-AD mice. Results are expressed as pg/ml of TNFα, IFNγ, IL12p70, IL6, IP10, MCP1, IL1β, IL10, IL4, and IL13. Statistical analysis of cytokine /chemokine profiles in plasma collected pre and 4 hrs post CpG ODN i.p. injection revealed significant differences **(** ***A*** **-** ***R*** **)**. See Results section for *p* values. An evident increase in most measured cytokines/chemokines was observed in both study groups. However, IL1β was detected at very low levels and no differences were observed between groups **(** ***G*** **,** ***P*** **)**. In addition, IL4 levels were near the limit of detection (data not shown). As expected, reduced cytokine levels were detected in plasma collected 2 weeks post CpG ODN injection **(** ***A*** **-** ***R*** **).** See Results section for *p* values. Data are presented as cytokine responses in plasma samples collected after the 1^st^ injection from 7 **(** ***A*** **-** ***I*** **)** and 11 **(** ***J*** **-** ***R*** **)** month old Tg-control and Tg-CpG ODN mice. No apparent differences in cytokine levels were observed between the groups at the time of sacrifice (data not shown). Overall, administration of CpG ODN was effective in inducing immunostimulatory response in the absence of excessive and chronic inflammation in both treatment groups.

Analysis was performed to assess whether administration of CpG ODN in 3xTg-AD mice was effective in inducing immunostimulatory response in the absence of any toxicity. Cytokine profiles in plasma collected 4 hrs after an i.p. injection of CpG ODN or saline were measured at monthly intervals, using the Th1/Th2 Luminex mouse cytokine/chemokine kit. Statistical analyses of cytokine profiles in plasma collected pre and post 1^st^ CpG ODN administration revealed significant differences as shown in Figure 17A-R. There was a noticeable increase in TNFα [7–20 months, two-tailed *t*-test (applies to all subsequent *t*-tests for cytokine responses), ***p = 0.0001, Figure 17A; 11–18 months, ***p = 0.0002, Figure 17J] and IFNγ plasma levels 4 hrs post CpG ODN administration compared to saline animals (7–20 months, *p = 0.017, Figure 17B; 11–18 months, *p = 0.01, Figure 17K). An evident increase in IL12p70 was observed in the 7–20 month study group (*p = 0.002, Figure 17C). IL12p70 cytokine was detected in low levels and no significant differences were observed between the treatment groups at various time points in the 11–18 months age group. Our subjects showed a strong induction of IL6 plasma levels (7–20 months, **p = 0.007, Figure 17D; 11–18 months, **p = 0.004, Figure 17M). In addition, higher peak plasma levels of IP10 (Interferon gamma-induced protein 1 also known as CXCL10), and MCP1 (monocyte chemotactic protein-1 also known as CCL2) chemokines were observed after CpG ODN injection in the 7–20 month study group (IP10, ***p < 0.0001, Figure 17E; MCP1, **p = 0.002, Figure 17F). Similar effects were seen in the 11–18 month study group (IP10, **p < 0.008, Figure 17N; MCP1, *p = 0.04, Figure 17O). IL1β elevation has been linked to induction of tau related pathology [41]. Of interest to us, IL1β, a potent inflammatory cytokine, was detectable at very low levels, and exhibited no significant changes over time between the groups (Figure 17G, P). Furthermore, IL10, a potent anti-inflammatory cytokine, was also significantly induced in response to stimulation with CpG ODN in both aged groups (7–20 months, **p = 0.002, Figure 17H; 11–18 months, *p = 0.01, Figure 17Q). Results for IL4 showed most measurements to be near the detection limit and no differences were observed between the groups (data not shown). There was a strong trend toward increase in IL13 plasma levels in our treated groups (7–20 months, *p* = 0.09, Figure 17I; 11–18 months, *p* = 0.1 Figure 17R). Separate plasma cytokines analysis were also performed in plasma samples collected 2 weeks after selected injections to evaluate the kinetic pattern of cytokine induction (Figure 17A-R). As expected, 2 weeks after the first injection, the levels of TNFα (7–20 months, ***p < 0.0001, Figure 17A; 11–18 months, ***p < 0.0001, Figure 17J), IFNγ (7–20 months, *p = 0.028, Figure 17B; 11–18 months, **p = 0.026, Figure 17K), IL12p70 (7–20 months, *p = 0.049, Figure 17C), IL6 (7–20 months, *p = 0.018, Figure 17D; 11–18 months, ***p < 0.0001, Figure 17M), IP10 (7–20 months, ***p < 0.0001, Figure 17E; 11–18 months, ***p < 0.0001, Figure 17N), MCP1 (7–20 months, **p = 0.026, Figure 17F; 11–18 months, **p = 0.008, Figure 17O), IL10 (7–20 months, **p = 0.006, Figure 17H; 11–18 months, ***p < 0.0001, Figure 17Q), IL13 (7–20 months, *p = 0.03, Figure 17I) were significantly lower than what was observed in plasma collected 4 hrs post 1^st^ CpG ODN administration. Since the levels of IL12p70 were limited and only slightly enhanced compared to saline treated animals in the 11–18 month study group, no difference was observed at the two week time point (Figure 17L). IL13 decreased 2 weeks later but did not reach statistical significance (11–18 month study group, Figure 17R). Similar cytokine responses were detected in plasma samples collected 4 hrs or 2 weeks after subsequent monthly injections.

Furthermore, no significant differences in cytokine levels were found between the groups at the time of sacrifice, 4 weeks after the last CpG ODN injection. The cytokine levels subsided over time (data not shown). Overall, TLR9 agonist CpG ODN elevated the levels of various Th1/Th2 cytokines-chemokines, but the levels were considerably lower than what was observed in previous reports with TLR4 agonist LPS [42,43]. Our findings demonstrate that stimulation of TLR9 signaling with CpG ODN seems to induce a suitable degree of innate immune stimulation that reduces the accumulation of AD related pathology, without producing excessive and sustained inflammation.

**Figure 18:**
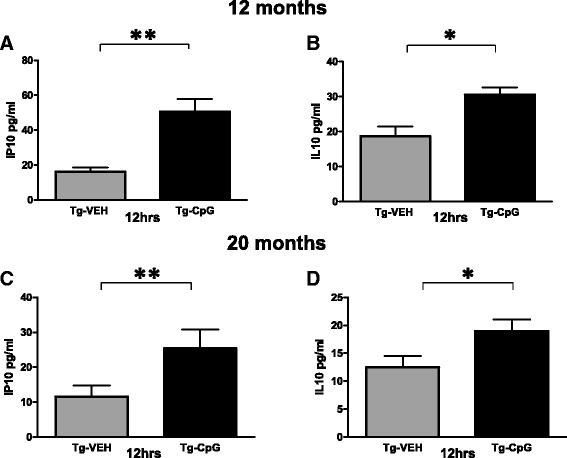
**Brain tissue cytokine/chemokine analysis (acute experiment).** Immunostimulatory responses to acute CpG ODN treatment were further characterized in brain tissue samples in 3xTg-AD mice. Unlike in plasma, only 2 cytokines were released at a detectable concentration. In both age cohorts, there were significant increases in IP10 (young, **p = 0.003; aged, **p = 0.007) **(** ***A*** **,** ***C*** **)** and IL10 (young, *p = 0.014; aged, *p = 0.04) **(** ***B*** **,** ***D*** **)** brain tissue cytokine levels 12 hrs post CpG ODN administration.

We next examined the plasma and brain cytokine responses to acute TLR9 signaling stimulation. Plasma cytokine profiles assessed at the time of sacrifice, 12 hrs after CpG ODN or saline i.p. administration, were comparable to the induction of plasma cytokines observed in our original long term study (data not shown). In the brain samples, only 2 cytokines were released in a detectable concentration. There was a significant increase in IP10 (two-tailed *t* test, young, **p = 0.003; aged, **p = 0.007, Figure 18A, C) and IL10 (young, *p = 0.014; aged, *p = 0.04, Figure 18B, D) brain tissue cytokine levels 12 hrs post CpG ODN administration in both age cohorts.

## Discussion

Immunomodulation has shown great promise as an AD therapy, at least in animal models, but major limitations must be overcome for greater clinical efficacy. Recently, two large phase III clinical trials using anti-Aβ antibodies, Bapineuzumab and Solunezumab, failed to show overall clinical improvement or any clear disease modifying results [44,45]. We suggest that for immunomodulation to be more successful, the methodology must show efficacy against all the key pathologies in AD: amyloid plaques, CAA, tau related pathology, and toxic oligomer species, with a few or no associated microhemorrhages [2,10].

In our initial studies, we utilized type B CpG ODN to stimulate innate immunity via TLR9 in the Tg2576 AD mouse model and showed this to be highly effective at reducing parenchymal and vascular amyloid burden, correlating with behavioral improvements [9]. However, there is growing recognition that AD therapy needs to also address tau related pathology and recent reports have shown that active and passive immunotherapeutic approaches can successfully reduce tau aggregates and improve cognition in mouse models of AD [10,46]. Our current findings demonstrate that stimulation of TLR9 signaling with CpG ODN has beneficial effects on both plaque and tangle pathologies without toxicity in 3xTg-AD mice, suggesting that stimulation of innate immunity has the possible advantage of concurrently addressing both of these fundamental lesions that characterize AD.

Experiments were designed to test the hypothesis that CpG ODN treatment can prevent the age-dependent accumulation of tau and Aβ when started prior to pathology development, as well as in established disease. Peripheral administration of CpG ODN led to a significant reduction in cortical and hippocampal amyloid burden and was effective against tau related pathology in both treatment groups. Immunohistochemistry revealed a reduction of several tau markers (MC1, PHF1 and AT8) in the CpG ODN-treated animals, whereas levels of total tau remained unchanged. These favorable treatment effects of CpG ODN were associated with an improvement in cognitive functioning as assessed by radial arm maze. Cognitive improvements were not confounded by any locomotor differences between the Tg mouse groups. A large body of evidence supports the concept that the accumulation of aggregated Aβ and tau species as toxic oligomers, correlates best with cognitive deficits, making oligomers attractive therapeutic targets [47,48]. The behavioral rescue observed in animals treated with CpG ODN was associated with a marked reduction in Aβ oligomers as documented by Western blot and ELISA. In addition, treatment with CpG ODN led to substantial reductions in soluble and total Aβ40 and Aβ42 levels. Levels of CP13 soluble tau levels (S1 fraction) were significantly decreased in mice treated from 11 to 18 months. A trend for reduced PHF1 reactive soluble tau was observed in the same treatment group. It is likely that the earlier stage CP13 phospho-tau aggregates were easier to clear than later stage PHF1 assemblies. No alteration in total tau levels was detected biochemically in our animals treated with CpG ODN, supporting the safety of this approach. To further confirm that this type of immunomodulation did not accelerate tau pathology, in contrast to some prior innate immunity stimulation approaches, additional biochemical analyses were performed. ELISA measurements did not reveal significant differences between the groups; hence, no increase in phospho-tau levels was noted in CpG ODN-treated mice. Our biochemical analyses did not demonstrate as robust CpG ODN treatment effect as observed with immunohistochemistry. One potential explanation for this discrepancy may be that histological analyses allowed measurement of reduced tau pathology immunoreactivity in individual cells, whereas homogenization of the whole brain hemisphere for biochemical analysis diluted any measurable changes in specific brain regions. It is possible that part of the tau pathology reduction effect we observed was secondary to a reduction in the Aβ pathology. However, since pathological tau was reduced in the older mouse group for which treatment began when some tau pathology was already present, we hypothesize that at least part of the tau pathology reduction was from a direct effect of CpG ODN stimulation. To definitively address this question, CpG ODN stimulation would need to be tested in a model with only tau related pathology, without concomitant Aβ pathology; these studies are underway.

Microglial responses, as well as potential signs of cerebral toxicity associated with the inflammatory potential of CpG ODN administration, were assessed at the end of the study. Microglia can develop a range of phenotypes broadly corresponding to a spectrum of the M1 state (classical activation) and the M2 state (alternative activation) [49,50]. The M1 state is associated with the release of pro-inflammatory cytokines while the M2 phenotype triggers anti-inflammation, promoting tissue repair [49,50]. The reduction of plaque and tangle pathology at the end of the study was paralleled by an overall reduction in the numbers of activated microglia, as evaluated by CD45 (M1 activation marker), CD206 (M2 activation marker) and CD11b (marker of both M1 and M2 activation) immunoreactivity in CpG ODN-treated animals. No group differences in the levels of Iba microgliosis (marker of both resting and activated microglia) and CNS astrocytosis were noted. We also did not observe any T-cell infiltrates in our CpG ODN group. These reductions in microglia markers in CpG ODN-treated animals at the end of long term treatment reflected the reduction of AD related pathology. We performed additional experiments to assess the effects of TLR9 signaling on the macrophage/microglia activation state and their ability to promote phagocytosis, on a more acute basis, in order to assess the mechanisms by which CpG ODN treatment could lead to AD pathology reduction. Our short term study was performed in two separate age cohorts of 3xTg-AD mice. Despite an overall reduction in CD45, CD11b and CD206 immunoreactivity due to reduced plaque load in the CpG ODN-treated mice at the end of our long term treatment, a transient increase in CD45, CD11b and CD206 microglial activation was observed 12 hrs after CpG ODN administration in both age groups. Clearance of AD brain pathology may depend on bone marrow-derived (BMD) macrophages rather than resident brain microglia [51-53]. It is likely that TLR9 stimulation with CpG ODN, which does not cross the BBB, involves targets in the periphery. Direct stimulation of resident microglia may also be possible to a limited extent as a result of BBB breakdown in the presence of AD pathology [54]. Double immunofluorescence using CD45 and CD206 markers (which are commonly expressed in BMD peripheral macrophages) revealed increased activation of microglial cells around amyloid plaques with co-localization of Aβ and these markers, suggesting that these cells were actively clearing Aβ deposits [51,55-57]. CD45 and CD206 positive cells were also noted to be increased in areas with high tau related pathology, but little or no direct co-localization was found. The mechanisms by which CpG ODN mediated stimulation of innate immunity could reduce tau pathology are unclear. Tau pathology has been demonstrated to spread cell to cell via tau oligomers in a prion-like manner [10]. It has also been shown that tau oligomers, the most toxic forms of aggregated tau, can activate microglial cells and increase their expression of scavenger receptor class A [58-60]. It is possible that TLR9 stimulation via CpG ODN can enhance the ability of microglia to inhibit this spread of tau related pathology, suggesting that induction of TLR9 signaling can be used to successfully target pathological tau in AD mouse models. Overall, our results suggest that CpG ODN treatment transiently stimulates a mixed microglia population with both M1 and M2 activation states to promote clearance of both Aβ and tau related pathology, along with tissue repair. However, long term treatment is associated with less microglia activation as the pro-inflammatory stimulus of AD related pathology is reduced.

In our prior study of CpG ODN treatment in Tg2576 AD model mice we found that the CpG ODN- treated mice had significantly higher levels of anti-Aβ autoantibodies at the end of the study [9]. We speculated that this may have been a minor part of the mechanism for the observed treatment effects. The contribution of this humoral response was judged to be small as the anti-Aβ titer, in absolute terms, was very low and it was found only at the end of treatment, not at earlier time points [9]. In the current study we did not find evidence that CpG ODN treatment increased autoantibodies to either Aβ or tau in 3xTg-AD mice. One possible reason for this difference is that 3xTg-AD mice have been suggested to be relatively immunodeficient compared to other AD Tg models [61]. In one of our previous active immunization studies where the same protocol was followed concurrently in both 3xTg-AD mice and Tg-SwDI AD model mice, the 3xTg mice had a substantially lower humoral immune response [19]. Hence, this difference in the 3xTg-AD mice provides a possible explanation for the discrepancy between our past and current findings.

Another drawback of current vaccination approaches is limited effectiveness against CAA. Current animal and human data suggest that CAA is more resistant to clearance compared to parenchymal amyloid and its removal may be associated with microhemorrhages [62-64]. Additionally, CAA may be involved in the amyloid related imaging abnormalities (ARIA) and/or vasogenic edema of some patients receiving Aβ-immunotherapy, complications that were not anticipated by numerous studies in transgenic mouse models [44,65]. Therefore, developing a therapy that is effective against CAA without inducing adverse reactions is of critical importance. The present data document a significant reduction in CAA burden in the absence of microhemorrhages after CpG ODN administration. Overall, our findings confirm that administration of CpG ODN was effective in reducing AD related pathology in the absence of encephalitis and without apparent autoimmune complications, further supporting the safety of this approach.

There is evidence that neuroinflammation can contribute to cognitive impairment and play a significant role in the disease progression [6,11,66]. Conversely, it is becoming increasingly recognized that tightly regulated stimulation of innate immunity processes and specific microglia activation can be neuroprotective depending on the stimulus and the environment [67]. Experimental evidence suggests that TLR signaling pathways may be involved in the clearance of Aβ deposits in the brain [12,68]. An in vitro study has shown that stimulation of microglia with TLR9 agonist CpG ODN attenuated the neurotoxic effect of Aβ42 oligomers [69]. We and others have demonstrated that microglial activation associated with TLR9 has benefits in amyloid depositing mice [9,69]. A recent study using the TLR4 agonist monophosphoryl lipid A (MPL) gave further evidence that TLRs can be a therapeutic target in AD [42]. However, only limited studies have been performed in tau mouse models. Treatment approaches that reduce amyloid pathology appear to accelerate tau deposition [11-16]. TLR4 ligand (LPS)-induced MAPT hyperphosphorylation and exacerbation of tau pathology has been well documented [16,39,40]. Additionally, proinflammatory cytokines, such as IL1β, have been shown to accelerate formation of NFTs [41]. A number of studies suggest that inflammation and altered microglial activation play a role in modulating hyperphosphorylation and aggregation of tau, via activation of tau specific kinases [16,40]. Conversely, our data clearly demonstrates that stimulation of innate immunity with TLR9 agonist CpG ODN can reduce both amyloid and tau related pathologies.

To evaluate the pattern of cytokine induction by CpG ODN we measured cytokine profiles in the plasma both 4 hrs and 2 weeks following injections. CpG ODN elevated the levels of both Th1/Th2 cytokines and chemokines, but the levels were substantially lower than those generated by LPS in previous studies [42,43]. Importantly, production of IL1β, which was shown to exacerbate tau pathology, was detected at very low levels, and no significant changes were observed between our groups over time [70]. There is also evidence that suggests an important role of MCP1 (CCL2) in AD. While MCP1 can enhance microglial Aβ degradation, both *in vivo* and *in vitro*, chronic expression of MCP1 has adverse effects on Aβ deposition [71,72]. Higher peak plasma levels of MCP1 chemokine were observed 4 hrs after CpG ODN injection in our study groups; however, the MCP1 levels declined over time. In addition, the levels of anti-inflammatory cytokine IL10, which induces the M2-alternative activated phenotype in microglia, were significantly increased after CpG ODN administration. IL10 over-expression has been shown to be beneficial in APP mice [73]. Furthermore, we show that peripheral administration of CpG ODN is not associated with excessive and sustained inflammation, which was confirmed by reduced cytokine levels measured 2 weeks post-injection.

Several reports have suggested a potential benefit of therapeutically targeting mononuclear cells to reduce AD related pathology [2,52]. Microglia lose their Aβ clearing capabilities as AD progresses [51,74,75]. Senescence of microglia function has been suggested to play a fundamental role in both AD and other neurodegenerative diseases [28,76]. Prior studies have also shown that microglia become significantly more efficient at Aβ uptake and degradation when stimulated with TLR agonists in vitro [77,78]. It has been reported that activation of microglia with TLR agonists can induce acidification of lysosomes, allowing efficient degradation of Aβ [75]. However, the relationship to tau pathology has yet to be studied. We hypothesize that our approach of immunomodulation can boost peripheral macrophages/microglia to specifically clear both species, enhancing pathological Aβ and tau trafficking to endosomes/lysosomes leading to more efficient degradation.

## Conclusions

The present study represents the first *in vivo* evidence that stimulation of TLR9 signaling with CpG ODN reduces behavioral deficits and is effective against all the major pathological hallmarks of AD in the absence of apparent toxicity. In addition, these beneficial effects of CpG ODN in 3xTg-AD mice were observed when treatment was initiated early in the course of disease as well once more advanced disease had set in. This suggests that the age related defects in immune cell function may be reversed by limited innate immunity stimulation via TLR9, even at later stages of disease. Any future clinical use of CpG ODN for AD would likely target a population of whom a substantial proportion will already have extensive pathology. Further studies using a well characterized non-human primate model of sporadic CAA, aged squirrel monkeys, are ongoing in our group and will provide essential preclinical evidence for CpG ODN as a disease modifying drug for AD in the setting of established pathology. Several CpG DNA drugs have shown favorable safety profiles in humans and have been tested in numerous clinical trials as anti-tumor, antimicrobial agents and as adjuvants in vaccines [8]. Our data are suggestive that stimulation of innate immunity has the potential to safely ameliorate all features of AD.

## References

[1] Prince M, Prina M, Geurchet M (2013). World Alzheimer Report 2013. Alzheimer’s Dis Int.

[2] Wisniewski T, Goni F (2014). Immunotherapy for Alzheimer’s disease. Biochem Pharmacol.

[3] Frackowiak J, Wisniewski HM, Wegiel J, Merz GS, Iqbal K, Wang KC (1992). Ultrastructure of the microglia that phagocytose amyloid and the microglia that produce beta-amyloid fibrils. Acta Neuropathol.

[4] Guillot-Sestier MV, Town T (2013). Innate Immunity in Alzheimer’s Disease: A Complex Affair. CNS Neurol Disord Drug Targets.

[5] Moraes CF, Lins TC, Carmargos EF, Naves JO, Pereira RW, Nobrega OT (2012). Lessons from genome-wide association studies findings in Alzheimer’s disease. Psychogeriatrics.

[6] Boutajangout A, Wisniewski T (2013). The innate immune system in Alzheimer’s Disease. Int J Cell Biol.

[7] Crack PJ, Bray PJ (2007). Toll-like receptors in the brain and their potential roles in neuropathology. Immunol Cell Biol.

[8] Vollmer J, Krieg AM (2009). Immunotherapeutic applications of CpG oligodeoxynucleotide TLR9 agonists. Adv Drug Deliv Rev.

[9] Scholtzova H, Kascsak RJ, Bates KA, Boutajangout A, Kerr DJ, Meeker HC, Mehta PD, Spinner DS, Wisniewski T (2009). Induction of Toll-like receptor 9 signaling as a method for ameliorating Alzheimer’s disease related pathology. J Neurosci.

[10] Boutajangout A, Wisniewski T (2014). Tau as a therapeutic target in Alzheimer’s disease. Gerontology.

[11] Lee DC, Rizer J, Hunt JB, Selenica ML, Gordon MN, Morgan D (2013). Review: experimental manipulations of microglia in mouse models of Alzheimer’s pathology: activation reduces amyloid but hastens tau pathology. Neuropathol Appl Neurobiol.

[12] Herber DL, Mercer M, Roth LM, Symmonds K, Maloney J, Wilson N, Freeman MJ, Morgan D, Gordon MN (2007). Microglial activation is required for Abeta clearance after intracranial injection of lipopolysaccharide in APP transgenic mice. J Neuroimmune Pharmacol.

[13] Henderson DM, Manca M, Haley NJ, Denkers ND, Nalls AV, Mathiason CK, Caughey B, Hoover EA (2013). Rapid antemortem detection of CWD prions in deer saliva. PLoS One.

[14] Liu Z, Condello C, Schain A, Harb R, Grutzendler J (2010). CX3CR1 in microglia regulates brain amyloid deposition through selective protofibrillar amyloid-beta phagocytosis. J Neurosci.

[15] Lee S, Varvel NH, Konerth ME, Xu G, Cardona AE, Ransohoff RM, Lamb BT (2010). CX3CR1 deficiency alters microglial activation and reduces beta-amyloid deposition in two Alzheimer’s disease mouse models. Am J Pathol.

[16] Bhaskar K, Konerth M, Kokiko-Cochran ON, Cardona A, Ransohoff RM, Lamb BT (2010). Regulation of tau pathology by the microglial fractalkine receptor. Neuron.

[17] Oddo S, Caccamo A, Shepherd JD, Murphy MP, Golde TE, Kayed R, Metherate R, Mattson MP, Akbari Y, LaFerla FM (2003). Triple-transgenic model of Alzheimer’s disease with plaques and tangles: intracellular Abeta and synaptic dysfunction. Neuron.

[18] Liu S, Breitbart A, Sun Y, Mehta PD, Boutajangout A, Scholtzova H, Wisniewski T (2014). Blocking the apolipoprotein E/amyloid β interaction in triple transgenic mice ameliorates Alzheimer’s disease related amyloid β and tau pathology. J Neurochem.

[19] Goni F, Herline K, Peyser D, Wong K, Ji Y, Sun Y, Mehta PD, Wisniewski T (2013). Immunomodulation targeting both Aβ and tau pathological conformers ameliorates Alzheimer’s Disease pathology in TgSwDI and 3xTg mouse models. J Neuroinflammation.

[20] Asuni A, Boutajangout A, Scholtzova H, Knudsen E, Li Y, Quartermain D, Frangione B, Wisniewski T, Sigurdsson EM (2006). Aβ derivative vaccination in alum adjuvant prevents amyloid deposition and does not cause brain microhemorrhages in Alzheimer’s model mice. Eur J Neurosci.

[21] McKee AC, Carreras I, Hossain L, Ryu H, Klein WL, Oddo S, LaFerla FM, Jenkins BG, Kowall NW, Dedeoglu A (2008). Ibuprofen reduces Abeta, hyperphosphorylated tau and memory deficits in Alzheimer mice. Brain Res.

[22] Asuni AA, Boutajangout A, Quartermain D, Sigurdsson EM (2007). Immunotherapy targeting pathological tau conformers in a tangle mouse model reduces brain pathology with associated functional improvements. J Neurosci.

[23] Morgan D, Gordon MN, Tan J, Wilcock D, Rojiani AM (2005). Dynamic complexity of the microglial activation response in transgenic models of amyloid deposition: implications for Alzheimer therapeutics. J Neuropathol Exp Neurol.

[24] Guerrero AR, Uchida K, Nakajima H, Watanabe S, Nakamura M, Johnson WE, Baba H (2012). Blockade of interleukin-6 signaling inhibits the classic pathway and promotes an alternative pathway of macrophage activation after spinal cord injury in mice. J Neuroinflammation.

[25] Cao T, Thomas TC, Ziebell JM, Pauly JR, Lifshitz J (2012). Morphological and genetic activation of microglia after diffuse traumatic brain injury in the rat. Neuroscience.

[26] Gordon S (2003). Alternative activation of macrophages. Nat Rev Immunol.

[27] He H, Xu J, Warren CM, Duan D, Li X, Wu L, Iruela-Arispe ML (2012). Endothelial cells provide an instructive niche for the differentiation and functional polarization of M2-like macrophages. Blood.

[28] Streit WJ, Braak H, Xue QS, Bechmann I (2009). Dystrophic (senescent) rather than activated microglial cells are associated with tau pathology and likely precede neurodegeneration in Alzheimer’s disease. Acta Neuropathol.

[29] Zotova E, Bharambe V, Cheaveau M, Morgan W, Holmes C, Harris S, Neal JW, Love S, Nicoll JA, Boche D (2013). Inflammatory components in human Alzheimer’s disease and after active amyloid-beta42 immunization. Brain.

[30] Yang J, Ji Y, Mehta P, Bates KA, Sun Y, Wisniewski T (2011). Blocking the apolipoprotein E/amyloid β interaction reduces fibrillar vascular amyloid deposition and cerebral microhemorrhages in TgSwDI mice. J Alzheimers Dis.

[31] Scholtzova H, Wadghiri YZ, Douadi M, Sigurdsson EM, Li Y, Quartermain D, Banerjee P, Wisniewski T (2008). A NMDA receptor antagonist leads to behavioral improvement and amyloid reduction in Alzheimer’s disease model transgenic mice shown by micro-magnetic resonance imaging. J Neurosci Res.

[32] Sadowski M, Pankiewicz J, Scholtzova H, Mehta P, Prelli F, Quartermain D, Wisniewski T (2006). Blocking the apolipoproteinE/Amyloid β interaction reduces the parenchymal and vascular amyloid-β deposition and prevents memory deficit in AD transgenic mice. Proc Natl Acad Sci U S A.

[33] Oddo S, Vasilevko V, Caccamo A, Kitazawa M, Cribbs DH, LaFerla FM (2006). Reduction of soluble Abeta and tau, but not soluble Abeta alone, ameliorates cognitive decline in transgenic mice with plaques and tangles. J Biol Chem.

[34] Sparks DL, Kryscio RJ, Sabbagh MN, Ziolkowski C, Lin Y, Sparks LM, Liebsack C, Johnson-Traver S (2012). Tau is reduced in AD plasma and validation of employed ELISA methods. Am J Neurodegener Dis.

[35] Goni F, Prelli F, Ji Y, Scholtzova H, Yang J, Sun Y, Liang FX, Kascsak R, Kascsak R, Mehta P, Wisniewski T (2010). Immunomodulation targeting abnormal protein conformation reduces pathology in a mouse model of Alzheimer’s disease. PLoS One.

[36] Washington PM, Morffy N, Parsadanian M, Zapple DN, Burns MP (2014). Experimental traumatic brain injury induces rapid aggregation and oligomerization of amyloid-beta in an Alzheimer’s disease mouse model. J Neurotrauma.

[37] Lesne SE, Sherman MA, Grant M, Kuskowski M, Schneider JA, Bennett DA, Ashe KH (2013). Brain amyloid-beta oligomers in ageing and Alzheimer’s disease. Brain.

[38] Pryor NE, Moss MA, Hestekin CN (2012). Unraveling the Early Events of Amyloid-beta Protein (Abeta) Aggregation: Techniques for the Determination of Abeta Aggregate Size. Int J Mol Sci.

[39] Lee DC, Rizer J, Selenica ML, Reid P, Kraft C, Johnson A, Blair L, Gordon MN, Dickey CA, Morgan D (2010). LPS- induced inflammation exacerbates phospho-tau pathology in rTg4510 mice. J Neuroinflammation.

[40] Kitazawa M, Cheng D, Tsukamoto MR, Koike MA, Wes PD, Vasilevko V, Cribbs DH, LaFerla FM (2011). Blocking IL-1 signaling rescues cognition, attenuates tau pathology, and restores neuronal beta-catenin pathway function in an Alzheimer’s disease model. J Immunol.

[41] Mrak RE, Griffin WS (2001). Interleukin-1, neuroinflammation, and Alzheimer’s disease. Neurobiol Aging.

[42] Michaud JP, Halle M, Lampron A, Theriault P, Prefontaine P, Filali M, Tribout-Jover P, Lanteigne AM, Jodoin R, Cluff C, Brichard V, Palmantier R, Pilorget A, Larocque D, Rivest S (2013). Toll-like receptor 4 stimulation with the detoxified ligand monophosphoryl lipid A improves Alzheimer’s disease-related pathology. Proc Natl Acad Sci U S A.

[43] Jaeger LB, Dohgu S, Sultana R, Lynch JL, Owen JB, Erickson MA, Shah GN, Price TO, Fleegal-Demotta MA, Butterfield DA, Banks WA (2009). Lipopolysaccharide alters the blood–brain barrier transport of amyloid beta protein: a mechanism for inflammation in the progression of Alzheimer’s disease. Brain Behav Immun.

[44] Salloway S, Sperling R, Fox NC, Blennow K, Klunk W, Raskind M, Sabbagh M, Honig LS, Porsteinsson AP, Ferris S, Reichert M, Ketter N, Nejadnik B, Guenzler V, Miloslavsky M, Wang D, Lu Y, Lull J, Tudor IC, Liu E, Grundman M, Yuen E, Black R, Brashear HR & Bapineuzumab 301 and 302 clinical trial investigators (including Wisniewski T) (2014). Two phase 3 trials of bapineuzumab in mild-to-moderate Alzheimer’s disease. N Engl J Med.

[45] Doody RS, Thomas RG, Farlow M, Iwatsubo T, Vellas B, Joffe S, Kieburtz K, Raman R, Sun X, Aisen PS, Siemers E, Liu-Seifert H, Mohs R (2014). Phase 3 trials of solanezumab for mild-to-moderate Alzheimer’s disease. N Engl J Med.

[46] Yoshiyama Y, Lee VM, Trojanowski JQ (2013). Therapeutic strategies for tau mediated neurodegeneration. J Neurol Neurosurg Psychiatry.

[47] Hefti F, Goure WF, Jerecic J, Iverson KS, Walicke PA, Krafft GA (2013). The case for soluble Abeta oligomers as a drug target in Alzheimer’s disease. Trends Pharmacol Sci.

[48] Castillo-Carranza DL, Lasagna-Reeves CA, Kayed R (2013). Tau aggregates as immunotherapeutic targets. Front Biosci (Schol Ed).

[49] Benarroch EE (2013). Microglia: Multiple roles in surveillance, circuit shaping, and response to injury. Neurol.

[50] Boche D, Perry VH, Nicoll JA (2013). Review: activation patterns of microglia and their identification in the human brain. Neuropathol Appl Neurobiol.

[51] Lai AY, McLaurin J (2012). Clearance of amyloid-beta peptides by microglia and macrophages: the issue of what, when and where. Future Neurol.

[52] Lampron A, Pimentel-Coelho PM, Rivest S (2013). Migration of bone marrow-derived cells into the central nervous system in models of neurodegeneration. J Comp Neurol.

[53] Jucker M, Heppner FL (2008). Cerebral and peripheral amyloid phagocytes–an old liaison with a new twist. Neuron.

[54] Sengillo JD, Winkler EA, Walker CT, Sullivan JS, Johnson M, Zlokovic BV (2013). Deficiency in mural vascular cells coincides with blood–brain barrier disruption in Alzheimer’s disease. Brain Pathol.

[55] Guillemin GJ, Brew BJ (2004). Microglia, macrophages, perivascular macrophages, and pericytes: a review of function and identification. J Leukoc Biol.

[56] Feng Y, Li L, Sun XH (2011). Monocytes and Alzheimer’s disease. Neurosci Bull.

[57] Durafourt BA, Moore CS, Zammit DA, Johnson TA, Zaguia F, Guiot MC, Bar-Or A, Antel JP (2012). Comparison of polarization properties of human adult microglia and blood-derived macrophages. Glia.

[58] Morales I, Jimenez JM, Mancilla M, Maccioni RB (2013). Tau oligomers and fibrils induce activation of microglial cells. J Alzheimers Dis.

[59] Sasaki A, Kawarabayashi T, Murakami T, Matsubara E, Ikeda M, Hagiwara H, Westaway D, George-Hyslop PS, Shoji M, Nakazato Y (2008). Microglial activation in brain lesions with tau deposits: comparison of human tauopathies and tau transgenic mice TgTauP301L. Brain Res.

[60] Ashe KH, Aguzzi A (2013). Prions, prionoids and pathogenic proteins in Alzheimer disease. Prion.

[61] St-Amour I, Pare I, Tremblay C, Coulombe K, Bazin R, Calon F (2014). IVIg protects the 3xTg-AD mouse model of Alzheimer’s disease from memory deficit and Abeta pathology. J Neuroinflammation.

[62] Pfeifer M, Boncristiano S, Bondolfi L, Stalder A, Deller T, Staufenbiel M, Mathews PM, Jucker M (2002). Cerebral hemorrhage after passive anti-Aβ immunotherapy. Sci.

[63] Wilcock DM, Jantzen PT, Li Q, Morgan D, Gordon MN (2007). Amyloid-beta vaccination, but not nitro-nonsteroidal anti-inflammatory drug treatment, increases vascular amyloid and microhemorrhage while both reduce parenchymal amyloid. Neurosci.

[64] Wilcock DM, Rojiani A, Rosenthal A, Subbarao S, Freeman MJ, Gordon MN, Morgan D (2004). Passive immunotherapy against Abeta in aged APP-transgenic mice reverses cognitive deficits and depletes parenchymal amyloid deposits in spite of increased vascular amyloid and microhemorrhage. J Neuroinflammation.

[65] Salloway S, Sperling R, Gilman S (2009). A phase 2 multiple ascending dose trial of bapineuzumab in mild to moderate Alzheimer disease. Neurol.

[66] Lampron A, Elali A, Rivest S (2013). Innate immunity in the CNS: redefining the relationship between the CNS and Its environment. Neuron.

[67] Schwartz M, Kipnis J, Rivest S, Prat A (2013). How do immune cells support and shape the brain in health, disease, and aging?. J Neurosci.

[68] Richard KL, Filali M, Prefontaine P, Rivest S (2008). Toll-like receptor 2 acts as a natural innate immune receptor to clear amyloid beta 1–42 and delay the cognitive decline in a mouse model of Alzheimer’s disease. J Neurosci.

[69] Doi Y, Mizuno T, Maki Y, Jin S, Mizoguchi H, Ikeyama M, Doi M, Michikawa M, Takeuchi H, Suzumura A (2009). Microglia activated with the toll-like receptor 9 ligand CpG attenuate oligomeric amyloid {beta} neurotoxicity in in vitro and in vivo models of Alzheimer’s disease. Am J Pathol.

[70] Ghosh S, Wu MD, Shaftel SS, Kyrkanides S, LaFerla FM, Olschowka JA, O’Banion MK (2013). Sustained interleukin-1beta overexpression exacerbates tau pathology despite reduced amyloid burden in an Alzheimer’s mouse model. J Neurosci.

[71] Yamamoto M, Horiba M, Buescher JL, Huang D, Gendelman HE, Ransohoff RM, Ikezu T (2005). Overexpression of monocyte chemotactic protein-1/CCL2 in beta-amyloid precursor protein transgenic mice show accelerated diffuse beta-amyloid deposition. Am J Pathol.

[72] Yamamoto M, Kiyota T, Walsh SM, Ikezu T (2007). Kinetic analysis of aggregated amyloid-beta peptide clearance in adult bone-marrow-derived macrophages from APP and CCL2 transgenic mice. J Neuroimmune Pharmacol.

[73] Kiyota T, Yamamoto M, Schroder B, Jacobsen MT, Swan RJ, Lambert MP, Klein WL, Gendelman HE, Ransohoff RM, Ikezu T (2009). AAV1/2-mediated CNS gene delivery of dominant-negative CCL2 mutant suppresses gliosis, beta-amyloidosis, and learning impairment of APP/PS1 mice. Mol Ther.

[74] Fiala M, Lin J, Ringman J, Kermani-Arab V, Tsao G, Patel A, Lossinsky AS, Graves MC, Gustavson A, Sayre J, Sofroni E, Suarez T, Chiappelli F, Bernard G (2005). Ineffective phagocytosis of amyloid-beta by macrophages of Alzheimer’s disease patients. J Alzheimers Dis.

[75] Majumdar A, Cruz D, Asamoah N, Buxbaum A, Sohar I, Lobel P, Maxfield FR (2007). Activation of microglia acidifies lysosomes and leads to degradation of Alzheimer amyloid fibrils. Mol Biol Cell.

[76] Streit WJ, Xue QS (2014). Human CNS immune senescence and neurodegeneration. Curr Opin Immunol.

[77] Iribarren P, Chen K, Hu J, Gong W, Cho EH, Lockett S, Uranchimeg B, Wang JM (2005). CpG-containing oligodeoxynucleotide promotes microglial cell uptake of amyloid beta 1–42 peptide by up-regulating the expression of the G-protein- coupled receptor mFPR2. FASEB J.

[78] Tahara K, Kim HD, Jin JJ, Maxwell JA, Li L, Fukuchi K (2006). Role of toll-like receptor signalling in Abeta uptake and clearance. Brain.

